# Functional neuroimaging effects of recently discovered genetic risk loci for schizophrenia and polygenic risk profile in five RDoC subdomains

**DOI:** 10.1038/tp.2016.272

**Published:** 2017-01-10

**Authors:** S Erk, S Mohnke, S Ripke, T A Lett, I M Veer, C Wackerhagen, O Grimm, N Romanczuk-Seiferth, F Degenhardt, H Tost, M Mattheisen, T W Mühleisen, K Charlet, N Skarabis, F Kiefer, S Cichon, S H Witt, M M Nöthen, M Rietschel, A Heinz, A Meyer-Lindenberg, H Walter

**Affiliations:** 1 Department of Psychiatry and Psychotherapy, Charité Universitätsmedizin Berlin, Campus Mitte, Berlin, Germany; 2 Division of Mind and Brain Research, Charité Universitätsmedizin Berlin, Campus Mitte, Berlin, Germany; 3 Analytical and Translational Genetics Unit, Department of Medicine, Massachusetts General Hospital and Harvard Medical School, Boston, MA, USA; 4 Department of Psychiatry, Psychosomatic Medicine, Psychotherapy, Goethe-University Frankfurt, Frankfurt, Germany; 5 Department of Genomics, Life and Brain Center, University of Bonn, Bonn, Germany; 6 Institute of Human Genetics, University of Bonn, Bonn, Germany; 7 Department of Psychiatry and Psychotherapy, Central Institute of Mental Health, Faculty of Medicine Mannheim, University of Heidelberg, Mannheim, Germany; 8 Department of Biomedicine, University of Aarhus, Aarhus, Denmark; 9 Institute of Neuroscience and Medicine, Research Centre Jülich, Jülich, Germany; 10 Division of Medical Genetics, Department of Biomedicine, University of Basel, Basel, Switzerland; 11 Department of Genetic Epidemiology in Psychiatry, Central Institute of Mental Health, Faculty of Medicine Mannheim, University of Heidelberg, Mannheim, Germany

## Abstract

Recently, 125 loci with genome-wide support for association with schizophrenia were identified. We investigated the impact of these variants and their accumulated genetic risk on brain activation in five neurocognitive domains of the Research Domain Criteria (working memory, reward processing, episodic memory, social cognition and emotion processing). In 578 healthy subjects we tested for association (i) of a polygenic risk profile score (RPS) including all single-nucleotide polymorphisms (SNPs) reaching genome-wide significance in the recent genome-wide association studies (GWAS) meta-analysis and (ii) of all independent genome-wide significant loci separately that showed sufficient distribution of all allelic groups in our sample (105 SNPs). The RPS was nominally associated with perigenual anterior cingulate and posterior cingulate/precuneus activation during episodic memory (*P*_FWE(ROI)_=0.047) and social cognition (*P*_FWE(ROI)_=0.025), respectively. Single SNP analyses revealed that rs9607782, located near *EP300*, was significantly associated with amygdala recruitment during emotion processing (*P*_FWE__(ROI)_=1.63 × 10^−4^, surpassing Bonferroni correction for the number of SNPs). Importantly, this association was replicable in an independent sample (*N*=150; *P*_FWE__(ROI)_<0.025). Other SNP effects previously associated with imaging phenotypes were nominally significant, but did not withstand correction for the number of SNPs tested. To assess whether there was true signal within our data, we repeated single SNP analyses with 105 randomly chosen non-schizophrenia-associated variants, observing fewer significant results and lower association probabilities. Applying stringent methodological procedures, we found preliminary evidence for the notion that genetic risk for schizophrenia conferred by rs9607782 may be mediated by amygdala function. We critically evaluate the potential caveats of the methodological approaches employed and offer suggestions for future studies.

## Introduction

Schizophrenia, a severe and often chronic disease that affects ~1% of the population, has one of the highest heritability estimates in psychiatry (80%).^1^ Genome-wide association studies (GWAS) have been uncovering an increasing number of common variants underlying disease susceptibility, promising valuable insights into pathogenic biological pathways. The largest GWAS to date including 36 989 cases and 113 075 controls identified 125 genetic loci (of which 108 were independent) associated with schizophrenia.^2^

It was suggested that investigating important neurocognitive domains implemented within specific brain circuits could be a promising way for biological psychiatry. The Research Domain Criteria (RDoC) approach^3, 4^ postulates five major domains (negative valence, positive valence, cognition, social processes and arousal/regulation) containing several subdomains of which many are relevant to schizophrenia. Following the RDoC rationale, in order to uncover biological mechanisms underlying mental illness, these domains warrant investigation at different units of analyses, including genetics and brain circuits. In the last years, imaging genetics studies investigated the impact of genetic risk variants on domain-related brain circuits (for overviews see refs 5, 6, 7). In our own previous work we were able to identify potential intermediate phenotypes^8, 9^ in five RDoC subdomains (working memory (WM), episodic memory, reward processing (RP), social cognition and emotion processing) that were modulated by risk variants within schizophrenia-associated variants (for example, *CACNA1C*, *ZNF804A*).^10, 11, 12, 13^ Importantly, several of these findings were successfully replicated in independent samples.^14, 15, 16^

However, all of these studies as well as the overwhelming majority of all other published imaging genetics investigations (see Stein *et al.*^17^ for a notable exception, though using structural neuroimaging) have been performed with single genetic variants and usually with only one neurocognitive paradigm.

A crucial next step is to comprehensively investigate the impact of the 108 independent schizophrenia-associated loci uncovered recently.^2^ One approach complementing single single-nucleotide polymorphism (SNP) analyses is the use of polygenic risk profiles. As the accumulation of genetic variants is known to form a substantial proportion of genetic susceptibility to psychiatric disease,^18^ Purcell *et al.*^19^ proposed the use of a polygenic risk profile score (RPS), a sum across risk alleles of multiple SNPs weighted by their effect size in an independent study. Employing RPS is considered a feasible approach to investigate the combined genetic impact of multiple variants in small samples.^20^ However, investigating the linear combination of several hundreds or thousands of variants may come at the expense of losing specificity; that is, RPS may obscure information conveyed by single or subsets of genes. Therefore, we decided to employ two complementary exploratory analyses. We investigated a range of promising intermediate phenotypes evoking activation of dedicated brain circuits relevant for schizophrenia (WM, episodic memory, RP, social cognition and emotion processing). These five neurocognitive subdomains cover four of the five general RDoC domains, that is, negative valence, positive valence, cognition and social cognition. First, we tested for association with an RPS including all SNPs reaching genome-wide significance (*P*<5 × 10^−8^; combined risk of 125 SNPs) in the recent GWAS meta-analysis. Second, we tested the effects of all genome-wide significant single variants that showed sufficient distribution of all allelic groups within our sample in order to identify contributions of specific variants. Results were assessed within task-specific target areas located within widespread brain circuits (Supplementary Figure S2).

## Materials and methods

### Subjects

A total of 578 German volunteers who never suffered from psychiatric disorder (evidenced by SCID-I)^21^ were recruited at Mannheim, Berlin and Bonn as part of an ongoing study on neurogenetic mechanisms of unipolar depression, bipolar disorder and schizophrenia (http://www.ngfn.de/en/ schizophrenie.html; http://www.sys-med.de/en/consortia/integrament/). *N*=333 participants had no lifetime family history of schizophrenia or an affective disorder, and *n*=245 subjects had at least one first-degree relative affected by schizophrenia (*n*=72), bipolar disorder (*n*=71) or depression (*n*=102). Affected index patients did not suffer from any other psychotic or affective disorder, and the investigated relatives had no family history of multiple different psychiatric diagnoses (for example, cases of both, affective and psychotic disorders). All subjects had grandparents of European origin. Following application of exclusion criteria *n*=472–509 subjects were included in the analyses of the respective tasks (see Supplementary Material for details). *N*=150 controls recruited as part of a study on the neurogenetic mechanisms of alcohol dependence in Berlin and Bonn (http://www.ngfn.de/en/alkoholabh__ngigkeit.html)^22^ served as replication sample. All participants never suffered from psychiatric disorder according to the Structured Clinical Interview of the Diagnostic and Statistical Manual of Mental Disorders (DSM-IV) Axis-I Disorders (SCID-I).^21^ Demographic characteristics of the respective subsamples are given in Supplementary Tables S1 and S2. The study was approved by local ethics committees of the universities of Heidelberg, Berlin and Bonn. All participants gave written informed consent to the study according to the Declaration of Helsinki.

### DNA extraction and genotyping

Ethylenediaminetetraacetic acid anticoagulated venous blood samples were collected from all individuals. Lymphocyte DNA was isolated using the Chemagic Magnetic Separation Module I (Chemagen, Baesweiler, Germany) according to the manufacturer’s recommendations. A genome-wide data set was generated at the Department of Genomics, Life & Brain Center, University of Bonn using Illumina’s Human610Quad, Human660W-Quad and Infinium PsychArray-24 BeadChips (Illumina, San Diego, CA, USA). Quality control and imputation were performed with standard parameters used by the Psychiatric Genetics Consortium (PGC) Statistical Analyses Group and RPS were calculated using methods described by Purcell *et al.*^19^ (see Supplementary Material for details).

### Functional imaging tasks

During functional magnetic resonance imaging (fMRI) subjects completed an associative episodic memory (EM) task requiring encoding, recall and recognition of face-profession pairs. The WM n-back task required continuous updating and retrieval of elements held in short-time memory. The RP monetary incentive delay task allowed the study of anticipation of monetary gains or losses. The Theory of Mind (ToM) task consisted of cartoon stories requiring subjects to take the protagonist’s perspective and judge changes in his/her affective states. The face-matching task (FMT) operationalized subjects’ implicit processing of negative emotions while viewing faces showing either fearful or angry expressions (for detailed descriptions see refs 10, 11, 12, 13 and Supplementary Material). All tasks were previously shown to robustly activate target structures and to possess excellent test–retest reliability in between-group designs.^12, 16, 23, 24^

### Imaging parameters

Blood-oxygen-level dependent fMRI was performed on three Siemens Trio 3 T MR-Tomographs at the Life and Brain Center of the University of Bonn, the Central Institute of Mental Health Mannheim, and the Charité-Universitätsmedizin Berlin. At all sites, identical sequences and scanner protocols were employed (EM: 33 slices, axially tilted (−30°), slice thickness 2.4 mm+0.6 mm gap, field of view (FOV) 192 mm, repetition time (TR) 1.96 s, echo time (TE) 30 ms, flip angle 80°, all other fMRI tasks: 28 slices, slice thickness 4 mm+1 mm gap, FOV 192 mm, TR 2 s, TE 30 ms, flip angle 80°). Quality-control measurements were conducted at all sites on every day of data collection according to a multicenter quality-assurance protocol, revealing stable parameters over time.^25^ To account for any variance related to differences across sites, the site was used as a covariate for all statistical analyses.

### Functional image processing

Image processing and statistical analyses were conducted using statistical parametric mapping methods as implemented in SPM8 (http://www.fil.ion.ucl.ac.uk/spm/software/spm8/). Scans were subjected to a strict quality assessment before inclusion into further analyses. Following data-quality assessment and application of general exclusion criteria (see above) *n*=472–509 subjects were included in the respective analyses (see Supplementary Figure 1 and Supplementary Methods for details). During pre-processing, images were realigned to a mean image (movement parameters confined to <3 mm translation and <1.7° rotation between volumes), slice-time-corrected, spatially normalized to a standard stereotactic space (a brain template created by the Montreal Neurological Institute) with a voxel size of 3 × 3 × 3 mm and smoothed with a 9 mm full width at half maximum Gaussian filter. A first-level fixed-effects model was computed for each participant and task. Regressors were created from the time course of the experimental conditions of interest per task and convolved with a canonical hemodynamic response function. Movement parameters, and for the ToM task also instructions and button presses, were included in the first-level models as covariates of no interest. For each subject, individual contrast images of the task effect (WM: 2-back>0-back; EM: memory>control; RP: anticipation of monetary win/loss>anticipation of neutral outcomes; ToM: mentalizing>control; FMT: faces>shapes) were subsequently entered into group statistics.

### Statistical group analyses

*RPS*: To test for genetic association with the intermediate phenotypes, the respective individual contrast images were analyzed using second-level multiple regression models for each task, including the RPS as regressor of interest, and age, sex, site, subgroup (no familial liability for psychiatric disorders, affected first-degree relative of patients with schizophrenia, bipolar disorder or depression), chip used for genotyping and the first three principal components (derived from a statistically independent set of common SNPs representing potential population stratification) as nuisance covariates. Effects of RPS on predefined regions of interest (ROIs) were assessed determining the familywise error (FWE)-corrected significance values of the maximally activated voxel within each ROI using undirected tests.

For the WM, EM and FMT tasks, ROIs were defined *a priori* using anatomical labels provided by the Wake Forest University Pick Atlas (www.fmri.wfubmc.edu/downloads). These were the bilateral dorsolateral prefrontal cortex (BA46 and BA9 with subtraction of medial voxels of BA9, see Esslinger *et al.*^11^ for details) for the WM task, the left and right hippocampus and perigenual anterior cingulate cortex (pgACC) for EM tasks and the bilateral amygdala, as well as the pgACC for the FMT (Supplementary Figure S2). As the AAL atlas does not provide anatomical labels for the ventral striatum, a spherical ROI was created for the RP task using a voxel located in the center of the ventral striatum (*x*=±9, *y*=11, *z*=−8) surrounded by a sphere of 10 mm. For the ToM task, four functional ROIs were created for those regions previously shown to be aberrantly activated in patients with schizophrenia (for example, Sugranyes *et al.*^26^; Walter *et al.*^27^) and to be genetically modulated.^13, 16^ As these regions were not covered by specific AAL ROIs, masks for the medial prefrontal cortex, bilateral temporal parietal junction (TPJ) and posterior cingulate cortex/precuneus (PCC/Pcu) were created based on coordinates reported by a meta-analysis on brain areas involved in ToM^28^ using the toolbox TWURoi (see Supplementary Material for details).

### Single SNP fMRI analyses

Comparable to RPS analyses, second-level multiple regression models were computed for each SNP and each task with number of minor alleles as regressor of interest, and age, sex, site, subgroup, genotyping chip and the first three principal components of potential population stratification as nuisance covariates. We excluded SNPs with less than 10 subjects in one allelic group, resulting in 105 independent analyses. Similar to RPS analyses, we extracted FWE-corrected *P*-values of the maximally activated voxel within each ROI for each SNP from an undirected test. These significance values were then Bonferroni-corrected for the number of autosomal SNPs tested, that is, 105 (*P*<4.76 × 10^−4^).

We used an undirected F-test in all analyses, as, for the investigated RPS and the vast majority of SNPs, we had no hypotheses about the directionality of effects. Associations with the intermediate phenotype could be represented by reduced or increased activation, being either indicative of dysfunction, inefficiency or compensatory resilience mechanisms.

## Results

### Polygenic RPSs

**Figure 1:**
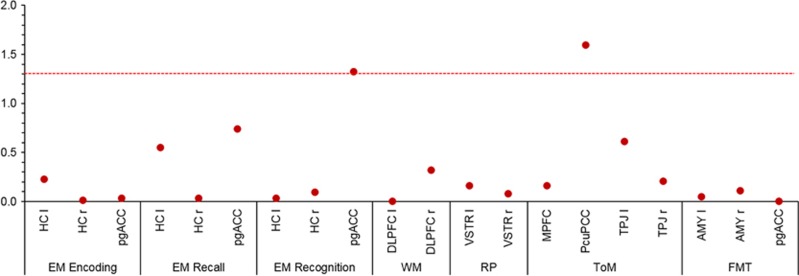
Results for the polygenic risk score (including all SNPs associated with schizophrenia at a genome-wide level, *n*=125) for regions of interest. Dashed line indicates –log10 *P*-value of familywise error correction for regions of interest in all tasks (*P*_FWE(ROI)_=0.05). *x* axis: region of interest, *y* axis: −log10 *P*-value for F-tests. AMY, amygdala; DLPFC, dorsolateral prefrontal cortex; EM, episodic memory; FMT, face-matching task; HC, hippocampus; l, left; pgACC, perigenual anterior cingulate cortex; MPFC, medial prefrontal cortex; Pcu/PCC, precuneus/posterior cingulate cortex; r, right; RP, reward processing; SNP, single-nucleotide polymorphism; ToM, Theory of Mind; TPJ, temporoparietal junction; VSTR ventral striatum; WM, working memory.

There were two significant associations between RPS and functional brain activation: accumulated genetic risk-predicted pgACC recruitment during EM recognition (*x*=−3, *y*=26, *z*=−11, F=13.72, *Z*=3.49, *P*_FWE__(ROI)_=0.047) and PCC/Pcu activity during ToM (*x*=−9, *y*=−55, *z*=22, F=13.90, *Z*=3.52, *P*_FWE__(ROI)_=0.025; Figure 1, Supplementary Figure S4).

### Genome-wide significant SNPs

**Figure 2:**
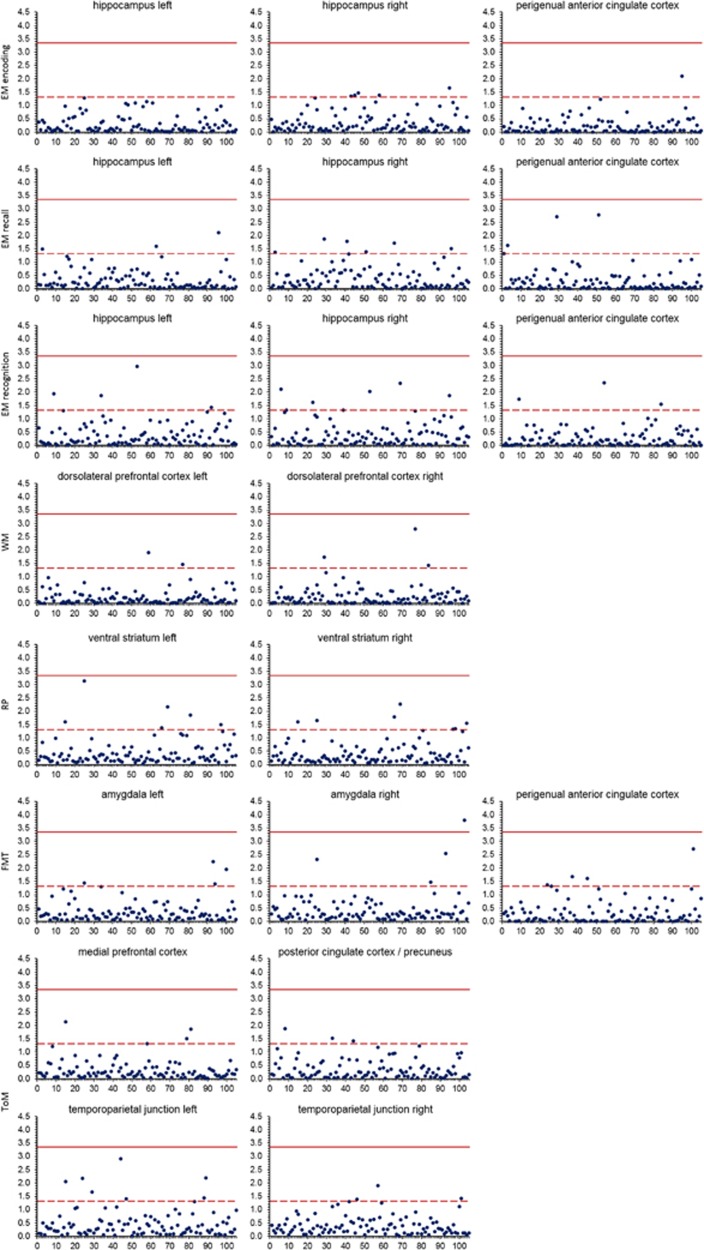
Results of analyses of single SNPs reaching genome-wide significance for association with schizophrenia for regions of interest in all tasks. Dashed line indicates –log10 *P*-value of familywise error correction for respective region of interest. Red line indicates –log10 *P*-value of correction for multiple tests (*P*=4 × 10^−4^). *x* axis: number of SNP, ordered by position within chromosome, *y* axis: −log10 *P*-value of F-tests. EM, episodic memory; FMT, face-matching task; RP, reward processing; ToM, Theory of Mind; SNP, single-nucleotide polymorphism; WM, working memory.

**Figure 3:**
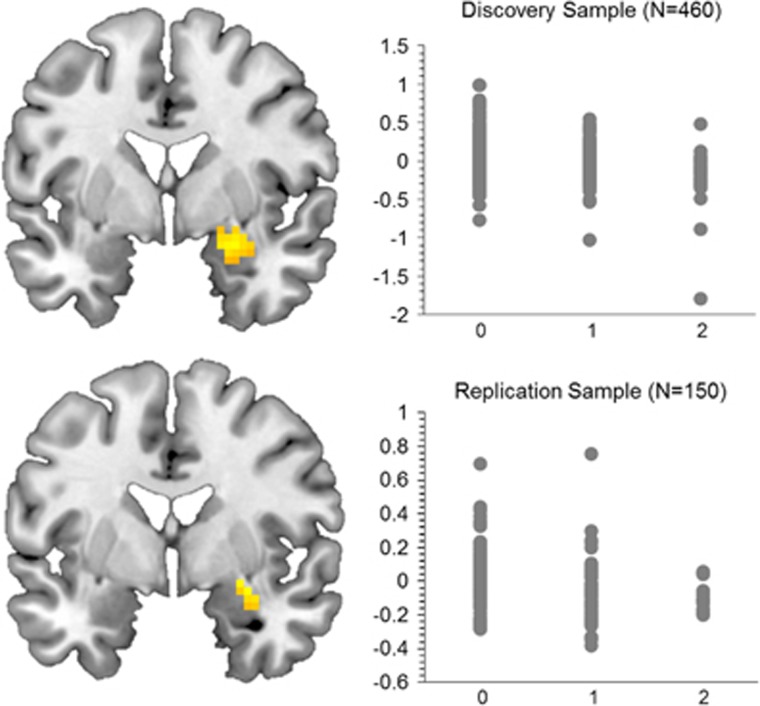
Effects of rs9607782 on right amygdala activity during implicit emotion processing. Results are *P*<0.05 FWE-corrected across the ROI. The effect in the discovery sample additionally withstood Bonferroni correction for the number of SNPs tested (*n*=105), that is, *P*_FWE_<0.0004. *N,* sample size; FWE, familywise error; ROI, region of interest; SNP, single-nucleotide polymorphism.

A median number of 4 (range 0–8) SNPs surpassed standard correction for multiple comparisons (*P*_FWE__(ROI)_<0.05) across ROIs (Figure 2, Table 1, Supplementary Table S3). These included replicated risk variants for schizophrenia associated with imaging phenotypes before, for example, rs11696094 (*ZNF804A*; ToM, RP), rs2007044 (*CACNA1C*; EM, RP), rs1702294 (*miR137*; EM) and rs9636107 (*TCF4*; EM). However, only one single SNP association withstood correction for multiple comparisons (number of tests per ROI), that is, rs9607782 (chr22, 41.40–41.68 Mb, hg19.37; located in an linkage disequilibrium (LD) block with *EP300*, *L3MBTL2, CHADL* and *RANGAP*) predicted right amygdala activation during the FMT (*x*=27, *y*=5, *z*=−17, F=21.45, *Z*=4.43, *P*_FWE__(ROI)_=1.63 × 10^−4^; Figure 3).

### Replication analysis

We could replicate the effect of rs9607782 on amygdala activation in an independent control sample using the identical FMT (*x*=30, *y*=−4, *z*=−11, F=10.94, *Z*=3.04, *P*_FWE__(ROI)_=0.025; Figure 3). In both samples amygdala activity decreased with increasing number of risk alleles for schizophrenia.

### Analyses of LD-independent non-schizophrenia-associated SNPs (*n*=105)

In order to assess the adequacy of correcting for the number of SNPs tested per ROI, we determined the number of significant effects of 105 common variants not significantly associated with schizophrenia on all neuroimaging phenotypes. Therefore, we generated a random set of 10 000 SNPs across the whole genome. From this set we randomly selected 105 variants with a *P-*value of >0.2 for association with schizophrenia and a minor allele frequency of >10%/<90% in the PGC_SCZ52 data set. We observed a median of three (range 0–7) significant hits applying a threshold of *P*_FWE__(ROI)_<0.05 across the respective ROIs. None of these associations withstood correction for the number of variants tested per ROI (Supplementary Figure S3). The difference between the number of statistically meaningful associations per ROI using schizophrenia-related and -unrelated SNPs was marginally significant (*P*=0.06 after 10 000 permutations; one-tailed *t*-test). However, overall the *P-*values observed with schizophrenia-associated variants were significantly lower than among associations with unrelated variants (*P*<0.005 after 10 000 permutations; one-tailed *t*-test; see Supplementary Material for details).

## Discussion

We investigated the impact of genetic risk for schizophrenia on a range of functional imaging phenotypes reliably activating distributed brain networks covering five RDoC subdomains with well-established relevance to the disease. We analyzed the effects of (i) a polygenic RPS and (ii) of 105 single SNPs genome-wide significantly associated with schizophrenia in face of benefits and drawbacks of both approaches. RPS offer assessment of accumulated genetic risk in a limited number of tests, but prohibit conclusions regarding specific contributions of SNPs and might conceal effects of some risk variants when combined with irrelevant signals. Single SNP analyses on the other hand have the disadvantage that the higher amount of tests (i) heightens the risk of false-positive results if not correctly accounted for (type I error) and (ii) increases the likelihood to find only those effects whose effect size is overestimated (winner’s curse).

The analysis of the polygenic RPS revealed two significant associations at a standard neuroimaging significance level (*P*_FWE__(ROI)_<0.05). The RPS predicted pgACC activity during EM recognition. pgACC recruitment was previously found to be modulated by genetic risk for schizophrenia during active EM retrieval. Furthermore, we found an association between RPS and PCC/Pcu activity during ToM. Activation of the PCC/Pcu, one of the crucial mentalizing areas, has been associated twice with a risk variant within ZNF804A (rs1344706) in high LD with a SNP included in the RPS (rs11693094; *R*^2^=0.84). However, it must be emphasized that these results would not withstand multiple comparison correction for the total number of ROI analyses across all tasks (*P*<0.0025, that is, *P*<0.05 FWE across 20 ROI analyses). With such a stringent threshold none of the above-mentioned results would remain significant and, in fact, one false-positive finding would be expected in 20 independent tests (at *P*=0.05).

As we investigated 105 independent SNPs, we strictly corrected for this number within each ROI. Only one association withstood correction for multiple testing: rs9607782 was associated with right amygdala activity during implicit emotion processing. To the best of our knowledge, this SNP was not previously investigated in imaging genetics studies. Rs9607782 is located in an LD block with *EP300, L3MBTL2, CHADL* and *RANGAP,* and is in high LD (*R*^2^=0.77) with a missense mutation in *EP300* (rs20551), suggesting functional relevance. Besides association with schizophrenia, mutations in the *EP300* gene are responsible for the Rubinstein-Taybi syndrome, a developmental disorder that includes intellectual disability, impulsivity, distractibility and mood instability.^29^ *EP300* encodes p300, a protein that functions as histone-acetyltransferase and is expressed in the brain in limbic and cortical regions. In mice, inhibition of p300 activity significantly impairs fear memory consolidation, and associated neural plasticity in the lateral amygdala.^30^ P300 inhibition further induced enhanced anxiety and mild cognitive impairment in a water maze task.^31^ Altered emotional significance detection and maladaptive appraisal have been associated with amygdala dysfunction in schizophrenia before.^32, 33, 34^ Thus, previous evidence supports the potential relevance of the observed association, particularly as we were able to replicate our result in an independent sample (Figure 3). This finding taps into the RDoC domains social cognition and negative valence. Importantly, the fMRI paradigm used to measure this potential intermediate phenotype is well qualified as an RDoC subdomain as it fulfills required criteria for good psychometric properties,^24^ association with psychopathology (association with schizophrenia was repeatedly shown on the behavioral and the brain level)^35, 36^ and heritability (with heritability estimates for emotion identification ranging between 0.21 and 0.43).^37, 38^

Other associations, although not surviving correction for multiple comparisons, include SNPs previously found to be related to imaging phenotypes from all RDoC domains assessed (see Table 1), for example, rs2007044 within *CACNA1C* was associated with hippocampal activity during EM,^10, 14, 15, 39^ but also with bilateral ventral striatal activation during RP. Rs11693094 within *ZNF804A* was associated with activity of ToM areas (medial prefrontal cortex, left temporal parietal junction) as shown before^13, 16^ and also with striatal activity. In addition, there were associations of variants within *TCF4* (previously found to have an impact on hippocampal volume)^40^ with hippocampal and pgACC activity during EM as well as with amygdala recruitment during implicit emotion processing. rs1702294 within *MIR137* (gene previously found to have an impact on frontalmediotemporal connectivity)^41, 42^ was associated with hippocampal activity, and rs2905426 within *NCAN* (previously associated with cortical thickness and folding in schizophrenia)^43, 44^ was associated with ventral striatal activation during RP.

All of these effects would stand out as significant hits in association studies on imaging phenotypes that typically apply a threshold of *P*_FWE__(ROI)_<0.05. However, an increasing number of statistical tests always comes at the expense of an increasing risk of type I error if not corrected for properly. In total, we found a median of 4 and up to 8 significant hits per ROI, which again, given a significance threshold of *P*<0.05, is compatible to chance findings in 105 tests. In fact, these numbers correspond well with those found by Sullivan who observed similar numbers in simulated genetic data containing no valid associations.^45^ In his data, an uncorrected significance level of *P*<0.05 yielded a proportion of false-positive findings of 96.8% (at least one positive finding in 968 of 1000 simulations). When Sullivan corrected for the number of SNPs he tested (which is in accordance to what we did) he still found a false-positive proportion of 31.4%.^45^ Thus, accounting for the number of SNPs may still be insufficient and additional correction for the number of ROIs could be necessary. Indeed, Sullivan reported that correction for the total number of independent tests resulted in the appropriate false-positive proportion of 5%. Application of such strict correction for our single SNP analyses (in our case *P*<2.3 × 10^−5^) would result in no finding being interpretable as statistically significant. On the other hand, the combination of FWE correction across ROIs with Bonferroni correction for the number of tests could be too conservative for fMRI data, taking into account that task-related ROI activation may be highly correlated, that is, is not truly independent. By testing associations of 720 SNPs not associated with schizophrenia with functional brain correlates of WM and implicit emotion processing Meyer-Lindenberg *et al.*^46^ showed that the false-positive proportion was no higher than 1.0–4.1% applying FWE correction alone. If this applies to our data, those SNPs surpassing the standard threshold of *P*_FWE__(ROI)_<0.05 may be true findings. Hence, to evaluate whether our findings contain true signal, we applied a similar strategy by testing the association of 105 SNPs not associated with schizophrenia or any mental function with our imaging phenotypes. Whereas the statistical difference for the total number of statistical meaningful associations among schizophrenia-related and -unrelated variants fell short of the significance threshold, we observed significantly higher probabilities for associations of disease-related than -unrelated variants. These results at least tentatively suggest further true signal in our data. Nevertheless, we believe that in face of the large amount of analyses we carried out in total, independent replication is essential and should be made mandatory in imaging genetics research, just as in psychiatric genetics research.

Our findings point to several issues relevant to the field of imaging genetics. It is foreseeable that larger genetic studies will discover more genome-wide significant genetic variants associated with psychiatric disorders. A brute force approach, that is, testing and correcting for all significantly associated variants known, will require a magnitude of statistical power difficult to achieve in functional imaging. There are three principal ways to respond to this conundrum. First, increase sample sizes (the mantra of genetics), for example, through consortial data accumulation worldwide. This is a reasonable path that has already been taken by the ENIGMA consortium^47^ to which we have contributed too.^17, 48^ However, costs and data availability limit this path and, in the foreseeable future, (relatively) large sample sizes will likely be restricted to structural and resting state data. The aggregation of task-related functional data will be even more difficult because of demands on harmonization of experimental procedures and psychometric task properties. Second, restrict analyses to theory-driven *a priori* hypotheses. This is formally correct but the possibility remains that ‘suitable’ hypotheses are established *post hoc* after extensive data mining. Hence, this is difficult to control without prior registration of hypotheses, in particular as more and more groups are performing genome-wide genotyping. That said, exploratory analyses should still be accepted if labeled appropriately and substantiated by replication studies. Third, and most forward-looking, the use of data reduction, feature selection and multivariate methods.^49^ Data reduction methods might include polygenic RPSs, gene set enrichment analyses or pathway analyses on the genetic side, and network analyses, independent component analyses, or graph theory on the imaging side. In addition, multivariate machine-learning methods might be useful to map high-dimensional genetic and neuroimaging data sets—this, however, also requires relatively large data sets.

Apart from these three principal approaches we would like to encourage the field to publish replication studies and in particular negative findings to enable future meta-analytical approaches. In addition, standards should be developed calling on imaging genetic studies to additionally publish whole-brain-effect sizes to facilitate replication and meta-analysis.

Our results suggest that risk scores aggregated across multiple independent loci may be less helpful than hoped for intermediate phenotype characterization. There is some evidence that brain effects are not as linear as associations with a (linear) polygenic RPS would imply.^50^ Further, it should be emphasized that comparable polygenic scores do not necessarily include the same combination of risk variants. Each individual will rather have a unique combination of risk variants, and it is thus not reasonable to assume that similar scores will have an impact on the same neural region or circuit. A possible solution would be genotyping a large sample of individuals and deeply phenotyping (using, for example, neuroimaging) a subset at the upper and lower tail of the distribution. This does not speak against the usefulness of RPS in general, but in intermediate phenotype research, more hypothesis-driven approaches to RPS definition may prove more fruitful.

Coming back to our own study presented here, we remind that our data are limited by the combined analyses of subjects with and without familial liability for psychiatric disorders. Still, all subjects had a negative lifetime history of psychiatric disorders as evidenced by a respective diagnostic interview and we accounted for subgroup status in all of our analyses.

Please note also that, in order to limit our variables on the imaging side, we restricted our analyses to regional effects using a ROI approach and did not test for other possible measures that focus on connectivity^11^ or network parameters.^51, 52^

In summary, although it unquestionably is a big challenge to acquire enough functional neuroimaging data for strict correction of both high-dimensional data sets (that is, imaging and genetics) using a brute force approach, we plead for the continuation of acquiring, analyzing and publishing imaging genetics results. These should include theory-driven restricted analyses, as well as stringent significance level adjustment and independent replication. Implementing these methodological requirements, our most robust finding was an association between a variant near *EP300* with amygdala function during implicit emotion processing, a finding that is supported by preclinical and clinical findings and points to a disease-relevant mechanism potentially mediating aberrant anxiety processing and/or fear memory.

## Figures and Tables

**Table 1:** SNPs surpassing FWE ROI-corrected threshold of *P*<0.05 for fMRI tasks

*ROI*	*Rank*	*SNP*	*Chr*	*Position*	*Gene*	*RA/NRA*	Z	P_FWE_*-ROI*	Vx	*MNI coords. (*x, y, z*)*			P*Sx*
*Episodic memory: encoding*													
HC l	*No significant findings*												
HC r	1	rs9636107	18	53 195 247–53 200 117	*TCF4*	GA	3.46	0.022	6	24	−37	4	3.34e−12
	2	rs12704290	7	86 403 226–86 459 326	*GRM3*	GA	3.31	0.035	1	15	−37	10	3.33e−10
	3	Chr6_84280274_D	6	84 279 922–84 407 274	*SNAP91*	I2D	3.24	0.042	2	36	−16	−17	8.15e−10
	4	rs11191419	10	104 585 135–104 956 335	*ARL3, AS3MT, C10orf32, CNNM2, CYP17A1, INA, NT5C2, PCGF6, PDCD11, SFXN2, TAF5, TRIM8, USMG5, WBP1L*	TA	3.23	0.042	1	33	−22	−17	6.20e−19
	5	rs11740474	5	153 671 057–153 688 217	*GALNT10*	TA	3.21	0.046	1	18	−13	−17	3.15e−8
pgACC	1	rs9636107	18	53 195 247–53 200 117	*TCF4*	GA	4.00	0.008	10	9	35	25	3.34e−12
													
*Episodic memory: recall*													
HC l	1	rs715170	18	53 769 014–53 804 154	*LOC100505474*	CT	3.73	0.008	8	−33	−10	−17	1.27e−8
	2	rs12421382	11	109 285 471–109 610 071	*C11orf87*	CT	3.38	0.026	3	−30	−37	−5	3.70e−08
	3	rs1498232	1	30 412 551–30 437 271	*—*	TC	3.29	0.032	3	−18	−13	−20	2.86e−9
HC r	1	rs9841616	3	181 023 585–181 205 585	*FXR1, DNAJC19, SOX2−OT*	TA	3.56	0.014	17	36	−13	−23	2.35e−8
	2	rs2973155	5	152 505 619–152 711 619	GRIA1*	CT	3.51	0.017	8	33	−7	−20	1.11e−10
	3	rs55661361	11	124 610 007–124 620 147	*ESAM, MSANTD2, NRGN, VSIG2*	GA	3.50	0.019	6	39	−22	−17	2.80e−12
	4	rs715170	18	53 769 014–53 804 154	*LOC100505474*	CT	3.31	0.032	2	39	−13	−20	1.27e−8
	5	rs3735025	7	137 039 644–137 085 244	*DGKI, PTN*	TC	3.22	0.042	2	36	−10	−26	3.28e−9
	6	rs1498232	1	30 412 551–30 437 271	*—*	TC	3.20	0.043	1	18	−28	−8	2.86e−9
pgACC	1	rs3735025	7	137 039 644–137 085 244	*DGKI, PTN*	TC	4.36	0.002	4	6	2	31	3.28e−9
	2	rs9841616	3	181 023 585–181 205 585	*FXR1, DNAJC19, SOX2−OT*	TA	4.32	0.002	26	−9	29	−5	2.35e−8
	3	rs1498232	1	30 412 551–30 437 271	*—*	TC	3.65	0.024	2	9	44	−2	2.86e−9
	4	rs4648845	1	2 372 401–2 402 501	*PLCH2*	TC	3.43	0.048	1	3	32	−2	8.70e−10
													
*Episodic memory: recognition*													
HC l	1	rs6984242	8	60 475 469–60 954 469	CA8*	GA	4.27	0.001	3	−33	−4	−26	5.97e−9
	2	rs10803138	1	243 503 719–243 612 019	*AKT3, SDCCAG8*	GA	3.64	0.012	2	−24	−10	−26	2.03e−8
	3	rs4391122	5	60 499 143–60 843 543	*ZSWIM6*	GA	3.60	0.014	3	−30	−22	−14	1.1e−14
	4	rs4523957	17	2 095 899–2 220 799	*SGSM2, SMG6, SRR, TSR1*	TG	3.30	0.037	1	−24	−4	−26	2.86e−10
	5	rs2909457	2	162 798 555–162 910 255	*DPP4, SLC4A10*	GA	3.22	0.049	1	−27	−19	−17	4.62e−8
HC r	1	rs2007044	12	2 321 860–2 413 760	*CACNA1C*	GA	3.93	0.005	16	36	−37	−8	3.22e−18
	2	rs1702294	1	98 374 984–98 559 084	*MIR137HG, MIR2682, MIR137, DPYD*	CT	3.78	0.008	6	36	−34	−8	3.36e−19
	3	rs6984242	8	60 475 469–60 954 469	CA8*	GA	3.71	0.009	6	30	−4	−26	5.97e−9
	4	rs9636107	18	53 195 247–53 200 117	*TCF4*	GA	3.61	0.014	6	30	−25	−8	3.34e−12
	5	rs4330281	3	17 221 366–17 888 266	*TBC1D5*	CT	3.43	0.025	1	24	−34	7	4.64e−9
	6	chr5_140143664_I	5	140 023 664–140 222 664	*AC005609.1, CD14, DND1, HARS, HARS2, IK, NDUFA2, PCDHA1, PCDHA10, PCDHA2, PCDHA3, PCDHA4, PCDHA5, PCDHA6, PCDHA7, PCDHA8, PCDHA9, TMCO6, WDR55, ZMAT2*	I12D	3.24	0.047	2	30	−13	−23	4.85e−8
	7	rs10803138	1	243 503 719–243 612 019	*AKT3, SDCCAG8, ANKRD63*	GA	3.21	0.047	1	24	−13	−23	2.03e−8
pgACC	1	rs7819570	8	89 340 626–89 753 626	*MMP16*	TG	4.15	0.005	29	−6	41	−5	1.22e−8
	2	rs10803138	1	243 503 719–243 612 019	*AKT3, SDCCAG8,*	GA	3.77	0.018	8	3	32	−14	2.03e−8
	3	rs190065944	15	78 859 610–78 859 610	*CHRNA5, CHRNA3, CHRNB4*	AG	3.66	0.029	4	3	26	−11	4.71e−8
													
*Working memory*													
DLPFC l	1	rs55833108	10	104 587 583–105 165 583	*ARL3, AS3MT, C10orf32, CNNM2, CYP17A1, INA, NT5C2, PCGF6, PDCD11, SFXN2, TAF5, TRIM8, USMG5, WBP1L*	TG	3.92	0.012	5	−33	44	37	2.23e−8
	2	rs2068012	14	30 189 985–30 190 316	*PRKD1*	CT	3.66	0.035	5	−42	26	34	1.41e−8
DLPFC r	1	rs2068012	14	30 189 985–30 190 316	*PRKD1*	CT	4.47	0.002	29	33	26	37	1.41e−8
	2	rs9841616	3	181 023 585–181** **205 585	*FXR1, DNAJC19, SOX2−OT*	TA	3.81	0.019	5	30	38	37	2.35e−8
	3	rs190065944	15	78 859 610–78 859 610	*CHRNA5, CHRNA3, CHRNB4*	AG	3.63	0.038	3	27	56	34	4.71e−8
													
*Reward processing*													
VSTR l	1	rs2535627	3	52 541 105–52 903 405	*GLT8D1, GNL3, ITIH1, ITIH3, ITIH4, MUSTN1, NEK4, NISCH, NT5DC2, PBRM1, SMIM4, SPCS1, STAB1, TMEM110, TMEM110-MUSTN1*	TC	4.13	0.001	20	−6	2	−8	4.26e−11
	2	rs2007044	12	2 321 860–2 413 760	*CACNA1C*	GA	3.46	0.007	35	−3	14	−5	3.22e−18
	3	rs56205728	15	40 566 759–40 602 237	*ANKRD63, PAK6 PLCB2*	AG	3.24	0.014	8	−18	14	−11	4.18e−9
	4	rs11693094	2	185 601 420–185 785 420	*ZNF804A*	CT	3.05	0.026	3	0	8	−8	1.53e−12
	5	rs2905426	19	19 374 022–19 658 022	*NCAN, HAPLN4, TM6SF2, SUGP1, MAU2, GATAD2A, TSSK6, NDUFA13, YJEFN3, CILP2, PBX4*	GT	2.97	0.032	3	−18	8	−5	3.63e−10
	6	rs55661361	11	124 610 007–124 620 147	*ESAM, MSANTD2, NRGN, VSIG2*	GA	2.82	0.042	1	−9	17	−5	2.80e−12
VSTR r	1	rs2007044	12	2 321 860–2 413 760	*CACNA1C*	GA	3.54	0.005	28	6	5	−8	3.22e−18
	2	rs55661361	11	124 610 007–124 620 147	*ESAM, MSANTD2, NRGN, VSIG2*	GA	3.16	0.016	5	12	20	−5	2.80e−12
	3	rs2535627	3	52 541 105–52 903 405	*GLT8D1, GNL3, ITIH1, ITIH3, ITIH4, MUSTN1, NEK4, NISCH, NT5DC2, PBRM1, SMIM4, SPCS1, STAB1, TMEM110, TMEM110-MUSTN1*	TC	3.13	0.022	33	15	11	−5	4.26e−11
	4	rs11693094	2	185 601 420–185 785 420	*ZNF804A*	CT	3.05	0.026	2	0	8	−8	1.53e−12
	5	rs1023500	22	42 315 744–42 361 344	*CENPM, CYP2D6, FAM109B, NAGA, NDUFA6, SEPT3, SHISA8, SMDT1, SREBF2, TCF20, TNFRSF13C, WBP2NL*	TC	3.06	0.028	1	18	8	−8	3.43e−8
	6	rs2053079	19	30 981 643–31 039 023	*ZNF536*	GA	2.85	0.046	2	12	8	1	4.49e−9
	7	rs2905426	19	19 374 022–19 658 022	*CILP2, GATAD2A, HAPLN4, MAU2, NCAN, NDUFA13, PBX4, SUGP1, TM6SF2, TSSK6*	GT	2.82	0.047	1	18	11	−5	3.63e−10
													
*Theory of Mind*													
MPFC	1	3rs11693094	2	185 601 420–185 785 420	*ZNF804A*	CT	3.81	0.007	5	−6	41	37	1.53e−12
	2	Rs256205728	15	40 566 759–40 602 237	*ANKRD63, PAK6, PLCB2*	AG	3.63	0.014	3	9	41	37	4.18e−9
	3	rs26193698	14	99 707 919–99 719 219	*BCL11B*	GA	3.41	0.031	2	−18	56	28	4.8e−9
	4	rs1112691419	10	104 585 135–104 956 335	*WBP1L, CYP17A1, C10orf32, AS3MT, CNNM2*	TA	3.25	0.049	1	18	44	34	6.20e−19
TPJl	1	rs133921127	6	73 132 701–73 171 901	RIMS1*	CT	4.29	0.001	26	−39	−58	34	2.69e−8
	2	rs7405404	16	13 728 459–13 761 359	ERCC4*	TC	3.90	0.006	11	−48	−67	22	1.01e−9
	3	rs75968099	3	36 843 183–36 945 783	*TRANK1*	TC	3.90	0.007	15	−45	−67	22	1.05e−13
	4	rs11693094	2	185 601 420–185 785 420	*ZNF804A*	CT	3.78	0.009	24	−57	−61	19	1.53e−12
	5	rs9841616	3	181 023 585–181 205 585	*FXR1, DNAJC19, SOX2−OT*	TA	3.54	0.022	2	−60	−37	4	2.35e−8
	6	rs9922678	16	9 875 519–9 970 219	*GRIN2A*	AG	3.37	0.037	1	−54	−40	25	1.28e−8
	7	rs12704290	7	86 403 226–86 459 326	*GRM3*	GA	3.36	0.039	1	−54	−70	19	3.33e−10
	8	rs12148337	15	70 573 672–70 628 872	TLE3*	TC	3.23	0.049	1	−54	−64	31	1.79e−8
TPJr	1	rs11139497	9	84 630 941–84 813 641	*TLE1*	AT	3.70	0.013	13	51	−37	25	3.61e−9
	2	rs7267348	20	48 114 136–48 131 649	*KCNB1, PTGIS*	CT	3.35	0.037	3	45	−64	19	4.56e−8
	3	chr7_2025096_I	7	1 896 096–2 190 096	*MAD1L1*	D/I3	3.35	0.042	2	57	−55	37	8.2e−15
PcuPCC	1	rs7523273	1	207 912 183–208 024 083	*C1orf132, CD46, CR1L*	AG	3.67	0.013	2	−6	−43	34	4.47e−8
	2	rs1501357	5	45 291 475–45 393 775	*HCN1*	CT	3.42	0.031	1	3	−46	22	5.05e−9
	3	rs1339227	6	73 132 701–73 171 901	RIMS1*	CT	3.35	0.038	4	−6	−58	43	2.69e−08
													
*Implicit emotion processing*													
Amy l	1	rs8082590	17	17 722 402–18 030 202	*ATPAF2, DRG2, GID4, LRRC48, MYO15A, RAI1, SREBF1, TOM1L2*	GA	3.48	0.006	27	−24	−4	−20	1.77e−8
	2	rs6065094	20	37 361 494–37 485 994	*ACTR5, PPP1R16B, SLC32A1*	GA	3.23	0.011	4	−30	−4	−14	1.46e−11
	3	rs2535627	3	52 541 105–52 903 405	*GLT8D1, GNL3, ITIH1, ITIH3, ITIH4, MUSTN1, NEK4, NISCH, NT5DC2, PBRM1, SMIM4, SPCS1, STAB1, TMEM110, TMEM110-MUSTN1*	TC	2.85	0.037	3	−24	−1	−20	4.26e−11
	4	Chr18_52749216_D	18	52 747 686–52 752 696	*TCF4*	I2D	2.81	0.040	1	−21	−7	−17	8.03e−11
	5	rs4391122	5	60 499 143–60 843 543	*ZSWIM6*	GA	2.74	0.050	1	−21	−4	−17	1.1e−14
Amy r	1	rs9607782	22	41 408 556–41 675 156	*CHADL, EP300, L3MBTL2*	AT	4.43	0.000	20	27	5	−17	2.07e−11
	2	rs8082590	17	17 722,402–18,030,202	*ATPAF2 DRG2 GID4 LRRC48 MYO15A RAI1 SREBF1 TOM1L2*	GA	3.70	0.003	44	30	−4	−20	1.77e−8
	3	rs2535627	3	52 541 105–52 903 405	*GLT8D1, GNL3, ITIH1, ITIH3, ITIH4, MUSTN1, NEK4, NISCH, NT5DC2, PBRM1, SMIM4, SPCS1, STAB1, TMEM110, TMEM110-MUSTN1*	TC	3.54	0.005	30	24	2	−20	4.26e−11
	4	rs8042374	15	78 803 032–78 926 732	*AC027228.1, AGPHD1, CHRNA3, CHRNA5, CHRNB4, IREB2, PSMA4*	AG	2.88	0.034	1	27	−1	−29	2.44e−13
pgACC	1	rs7267348	20	48 114 136–48 131 649	*KCNB1, PTGIS*	CT	4.33	0.002	16	−15	44	13	4.56e−8
	2	rs10043984	5	137 598 121–137 750 021	*CDC25C, CTNNA1, EGR1, ETF1, FAM53C, GFRA3, HSPA9, KDM3B, REEP2*	TC	3.67	0.021	4	0	26	−5	1.09e−8
	3	Chr6_84280274_D	6	84 279 922–84 407 274	*SNAP91*	I2D	3.67	0.025	16	0	44	−14	8.15e−10
	4	rs75968099	3	36 843 183–36 945 783	*TRANK1*	TC	3.51	0.042	1	−15	44	−5	1.05e−13
	5	rs832187	3	63 792 650–64 004 050	*ATXN7, C3orf49, PSMD6, THOC7*	CT	3.46	0.048	1	0	17	28	1.43e−8

Abbreviations: A, adenine; BP, base pair; C, cytosine; Chr, chromosome; dir, direction of effect (−, decreased activation with increasing number of risk alleles; +, increased activation with increasing number of risk alleles); fMRI, functional magnetic resonance imaging; Freq RA, frequency of risk allele in the present sample; G, guanine; HC, hippocampus; L, left; MNI coords., MNI coordinates of peak activation in mm; MPFC, medial prefrontal cortex; NRA non-risk allele; PcuPCC, precuneus/posterior cingulate cortex; pgACC, perigenual anterior cingulate cortex; *P*_FWE_-ROI, familywise error corrected *P*-value within region of interest; R, right; RA, risk allele; ROI, region of interest; SNP, single-nucleotide polymorphism; T, thymine; TPJ, temporal parietal junction; Vx, number of voxels in significant cluster.

Single SNP fMRI analysis of variants that have been associated with schizophrenia at a genome-wide significant level. Positions from hg19 build 37; *P-*values for association with schizophrenia (pSx) from combined sample of ref. 2. Genes were identified using Ricopili (data set: PGC_SCZ52_may13). All genes located within regions containing variants in linkage disequilibrium of *R*^2^⩾0.6 with the SNP associated with schizophrenia^2^ are reported. Where chromosomal positions do not contain a gene, the nearest genes within a ±500 kb window is listed (indicated by *).
