# Supplementary material for: Functional neuroimaging effects of recently discovered genetic risk loci for schizophrenia and polygenic risk profile in five RDoC subdomains
Source: Transl Psychiatry. 2017 Jan 10;7(1):e997–. doi: 10.1038/tp.2016.272 (PMC5545733; doi:10.1038/tp.2016.272)
Supplement: Supplementary Information [file tp2016272x1.docx]

**Supplementary Material**

Functional neuroimaging effects of recently discovered genetic risk loci for schizophrenia and polygenic risk profile in five RDoC-subdomains

S Erk, MD, PhD; S Mohnke, PhD.; S Ripke, MD, PhD; TA Lett, PhD; IM Veer, PhD; C Wackerhagen, Dipl.-Psych; O Grimm, MD; N Romanczuk-Seiferth, PhD; F Degenhardt, MD; H Tost, MD, PhD; M Mattheisen, MD; TW Mühleisen, PhD; K Charlet, PhD; N Skarabis, MA; F Kiefer, MD, PhD; S Cichon, PhD; SH Witt, PhD; MM Nöthen, MD, PhD; M Rietschel, MD, PhD; A Heinz, MD, PhD; A Meyer-Lindenberg, MD, PhD; H Walter, MD, PhD

This file includes:

Supplementary Methods

Imputation and generation of polygenic scores 2

Polygenic risk profile score prediction 2

Functional imaging tasks 3

Computation of literature based probabilistic ROIs for the ToM task 5

Permutation tests 6

Quality control procedures 7

Supplementary Table S1: Sample characteristics 8

Supplementary Table S2: Replication sample characteristics 9

Supplementary Table S3: Logarithmized significance values for effects of
single SNPs on functional ROIs 10

Supplementary Figure S1: Subject exclusion criteria 13
Supplementary Figure S2: Regions of interest 14

Supplementary Figure S3: Effects of SNPs not associated with
schizophrenia on regional brain activation 15

Supplementary Figure S4 18

References 19

**Supplementary Methods**

Imputation and generation of polygenic scores: Quality control and imputation were performed with standard parameters used by the PGC Statistical Analysis Group. The quality control parameters for retaining SNPs and subjects were: SNP missingness<0.05 (before sample removal); subject missingness<0.02; autosomal heterozygosity deviation (|Fhet|<0.2); SNP missingness<0.02 (after sample removal); and SNP Hardy-Weinberg equilibrium (p>10^−6^ in controls). After Quality Control and alignment to the imputation reference there were 315,628 common SNPs left for pre-phasing / imputation, implemented in IMPUTE2/SHAPEIT (chunk size of 3 Mb and default parameters).^1, 2^ The imputation reference set consisted of 2 186 phased haplotypes from the full 1000 Genomes Project dataset (August 2012, 30 069 288 variants, release “v3.macGT1”). After imputation, we identified SNPs with very high imputation quality (INFO>0.8) and low missingness (<1%) for further quality control. After linkage disequilibrium pruning (r^2^>0.02) and frequency filtering (MAF>0.05), there were 70 609 autosomal SNPs. This SNP set was used for robust relatedness testing and population structure analysis. Relatedness testing was done with PLINK ^3^ and pairs of subjects with $\hat{\pi}$>0.2 were identified and one member of each pair removed at random. Principal component estimation was done with the same collection of autosomal SNPs. We tested the first 20 principal components for phenotype association (using logistic regression with study indicator variables included as covariates) and evaluated their impact on the genome-wide test statistics using λ.^4^ The first three principal components were included in all association analyses.

Polygenic risk profile score prediction: As training dataset we took genome wide clumped results from ^5^ (see there for details). In order to get a highly informative SNP set with as little statistical noise as possible, we excluded uncommon SNPs (MAF < 10%), low-quality variants (imputation INFO < 0.9), indels, and SNPs in the extended MHC region (chr6:25-34 Mb). We then LD pruned and “clumped” the data, discarding variants within 500 kb of, and in r^2^ ≥ 0.1 with, another (more significant) marker. We performed RPS of our target individuals as originally described for two distinct p value thresholds (5x10^-8^, 0.05), multiplying the logistic regression ƅ (i.e., the natural log of the odds ratio) of each variant by the imputation probability for the risk allele in each individual. The resulting values were summed over each individual, so that each individual had a whole genome RPS for further analysis. The scores were normalized for all subsequent analyses.

Functional imaging tasks:

*Episodic memory (EM)*: During fMRI scanning subjects completes an episodic memory task that was part of three consecutive memory tasks, i.e. encoding, recall and recognition of face-profession pairs (for a detailed description, see ^6^). This task provides stable and reliable hippocampal activation with excellent test-retest reliability.^7^ During recall, the task focused on here, faces were presented together with the question whether the depicted person had to complete apprenticeship or academic studies in order to qualify for the respective profession that had to be learned during encoding. Subjects had to indicate by button press, which qualification was correct. In the control condition, subjects had to assess whether the left or the right ear of different head contours was larger. The task consisted of 4 blocks of 4 faces (a 6s) and 4 blocks of 6 head contours (a 4s) each. Each block lasted 24s.

*Working memory:* During this n-back task ^8^ subjects viewed a series of digits (1-4) presented sequentially for 500ms (ISI 1500ms). In the control condition (0-back) subjects had to press one of four buttons corresponding to the digit currently presented. In the working memory condition (2-back) they had to react to the digit seen two instances before the present digit. Four blocks of each condition were presented in alternation. Each condition had a duration of 30s and consisted of 15 trials.

*Reward processing:* In a well-established monetary incentive delay task subjects were asked to respond as fast as possible with a button press to a brief flashing of the screen. Different arrows shown before the flash indicated one of the four experimental conditions: (1) win: subjects could win 2€ if they responded fast enough (vertical arrow pointing upwards), (2) loss avoidance: subjects would lose 2€ if they responded too slow (vertical arrow pointing downwards), (3) verbal feedback: subjects received feedback on the speed of their reaction that did not have monetary consequences (double-headed vertical arrow), and (4) neutral: neither a screen flash, nor verbal feedback or monetary consequences followed a double-headed horizontal arrow. At the end of each trial, the balance of money lost and won so far was shown. A total of ten trials per condition were presented in pseudo-randomized order. Reaction time thresholds were adaptively adjusted to individual response times (i.e. a 5% increase after slow responses and a 5% decrease after fast responses). The task was shown to reliably activate striatal target structures.^9^

*Theory of Mind (ToM)*: This task consisted of 16 cartoon stories (8 per condition) comprising 3 images each. In the ToM condition subjects had to take the perspective of the protagonist in short cartoon stories and judge changes in his/her affective states (better, worse, equal compared to the preceding picture). In the control condition, subjects were asked to detect changes in the number of depicted living beings (more, less, equal than in the preceding pictures). Trials of both conditions were presented in alternation. This task was previously shown to robustly activate the mentalizing network and to possess excellent test-retest-reliability.^10-12^

*Face matching task:* During one trial subjects were either shown emotional faces (angry or fearful; emotional condition) or geometrical shapes (control condition). There were always two faces or forms at the bottom of the screen and one at the top. Subjects had to match those faces or forms shown at the bottom that matched the one shown above. There was a total of eight blocks (four blocks per condition), lasting 30s and consisting of six trials. This implicit emotion processing task robustly engages amygdala activity in between group designs.^13^

Computation of literature based probabilistic ROIs for the ToM task:

We decided to use functional regions of interest (ROIs) for analyses of the ToM task, because mentalizing regions cannot readily be assigned to separate anatomical areas (e.g. the TPJ includes parts of multiple temporal and parietal regions, see below) and smaller ROI size was desired to enhance specificity and sensitivity of analyses. Thus, a priori ROIs for small volume alpha error adjustment were created based on functional findings of brain areas consistently reported to be involved in ToM. These were the medial prefrontal cortex (MPFC), bilateral temporal junction (TPJ) and posterior cingulate cortex / precuneus (PCC/Pcu). ROI computation was carried out using the tool TWURoi, which is based on the procedure described by Schubert et al. and involved the following steps:

First, we created anatomical ROIs for the four regions using the AAL brain atlas. The MPFC ROI comprised the bilateral medial and superior frontal gyri, the bilateral TPJ ROIs were composed of the middle and superior temporal, angular and supramarginal gyri of the respective hemisphere, while the PCC/Pcu ROI encompassed the bilateral posterior cingulate cortex and precuneus.

Second, spatial coordinates for functional ROIs were taken from the meta-analysis on brain areas involved in mentalizing by Van Overwalle.^14^ Since this paper only listed coordinates for the MPFC and TPJ, although the PCC/Pcu can be reliably counted to the mentalizing network,^15^ coordinates for the PCC/Pcu were retrieved from the individual studies included in the meta-analysis by Van Overwalle.^14^

Third, based on this data set, we created the ROIs in a three-step process:^16^

(1) The probability that a voxel at a given position within an anatomical ROI showed neural activity regarding the corresponding literature was estimated by calculating a 3D normal (Gaussian) distribution G(x, y, z) as follows:^17^

where C is the covariance matrix for all coordinate triples x, y, z from the underlying literature and x, y, z are the mean values of the x, y, and z coordinates, respectively.^18^

(2) The outer limits of the finally used ROI were defined by (a) the outer limits of the anatomical ROI and (b) a threshold of 2 standard deviations of the resulting 3D distribution.

(3) Finally a binary mask including all voxels spatially within these boundaries was formed.

Specifically for spatially extended anatomical ROIs containing probably different functional subregions, this procedure leads to a spatially reduction to design relevant coordinates within these ROIs. The final volumes of the employed ROIs were as follows: MPFC: 545 voxels, left TPJ: 604 voxels, right TPJ: 685 voxels, PCC/Pcu: 692 voxels (see also Supplementary Figure 2).

Permutation tests: Permutation tests were carried out in order to assess differences between (1) the total number of significant associations with functional imaging phenotypes of schizophrenia-related and -unrelated variants, as well as (2) the probability values observed in either analysis. Permutation tests were perfo0rmed using Matlab 8.3 (Mathworks, Natick, Massachusetts, USA) using the function ‘mult_comp_perm_t1’ (accessible at: <http://www.mathworks.com/matlabcentral/fileexchange/29782-mult-comp-perm-t1-data-n-perm-tail-alpha-level-mu-reports-seed-state->). Paired t-tests were employed to assess differences between analyses with schizophrenia-related and -unrelated SNPs. Since we hypothesized an enrichment of meaningful signal among SNPs associated with schizophrenia, we used one-tailed tests. A total of 10.000 permutations were performed per analysis. In the first analysis the total number of significant hits per ROI (n=20 values for each SNP set) were compared (t=1.696, p=.061). In the second analysis all log p values observed for any association between SNPs and imaging phenotype were compared (n=2.100 values for each SNP set; n=105 associations in 20 ROIs; t=2.55, p=.005).

Quality control procedures:

Phantom measurements were conducted at all sites on every day of data collection with similar scanning sequences and the same head coils as the used in the main study. Phantom data were analysed with respect to signal and fluctuation over time according to the multi-center quality assurance protocol by Friedman and Glover.^19^ This revealed stable parameters over time. Scans were further inspected with respect to anatomical abnormalities, susceptibility artefacts and acquisition artefacts (e.g. slice positioning, spikes). Synchronicity between presentation and scanning was verified, movement parameters were confined to <3mm translation and <1.7° rotation between volumes and normalization success was confirmed.

**Supplementary Table S1:** Sample characteristics

|  | **Episodic memory** | | | **Working memory** | **Reward processing** | **Theory of Mind** | **Implicit emotion processing** |
| --- | --- | --- | --- | --- | --- | --- | --- |
|  | **Encoding** | **Recall** | **Recognition** |  |  |  |  |
| Subjects (NFR/FR) | 280/192 | 285/195 | 287/195 | 296/213 | 284/198 | 281/213 | 271/208 |
| Site (Bonn/Mannheim/Berlin) | 206/130/136 | 214/129/137 | 214/130/138 | 211/147/151 | 206/135/141 | 209/137/148 | 189/143/147 |
| Sex (male/female) | 195/277 | 202/278 | 203/279 | 215/294 | 204/278 | 209/285 | 202/277 |
| Handedness (right/left/both)* | 425/35/10 | 431/36/11 | 431/37/12 | 458/37/12 | 434/34/12 | 446/35/11 | 431/34/12 |
| Age (years, M±SD) | 32.2±10.4 | 32.3±10.4 | 32.4±10.4 | 32.3±10.4 | 32.3±10.4 | 32.2±10.4 | 31.9±10.2 |
| Educational years (M±SD) | 15.5±2.5 | 15.6±2.5 | 15.6±2.5 | 15.5±2.5 | 15.5±2.5 | 15.5±2.5 | 15.5±2.5 |

N sample size; NFR no familial risk for psychiatric disorders; FR familial risk for affective or psychotic disorders; Handedness was defined using the Edinburgh Handedness Inventory (Oldfield, 1971); M mean; SD standard deviation; *data missing for 2 subjects

**Supplementary Table S2:** Replication sample characteristics

|  | **Implicit emotion processing** |
| --- | --- |
|  |  |
| Site (Bonn/Berlin) | 61/89 |
| Sex (male/female) | 63/87 |
| Handedness (right/left/both) | 150/0/0 |
| Age (years, M±SD) | 40.5±12.8 |

Handedness was defined using the Edinburgh Handedness Inventory (Oldfield, 1971); M mean; SD standard deviation

**Supplementary Table S3:** Logarithmized significance values for effects of single SNPs on functional ROIs

|  |  | **EM encoding** | | | **EM recall** | | | **EM recognition** | | | **WM** | | **RP** | | **FMT** | | | **ToM** | | | |
| --- | --- | --- | --- | --- | --- | --- | --- | --- | --- | --- | --- | --- | --- | --- | --- | --- | --- | --- | --- | --- | --- |
| **# in figure** | **SNP** | **Hi L** | **Hi R** | **pgACC** | **Hi L** | **Hi R** | **pgACC** | **Hi L** | **Hi R** | **pgACC** | **DLPFC L** | **DLPFC R** | **VStr L** | **VStr R** | **AMY L** | **AMY R** | **pgACC** | **DMPFC** | **Pcu/PCC** | **TPJ L** | **TPJ R** |
| 1 | rs4648845 | 0.367 | 0.483 | 0.198 | 0.150 | 0.027 | 1.320 | 0.661 | 0.332 | 0.008 | 0.050 | 0.002 | 0.171 | 0.123 | 0.465 | 0.267 | 0.289 | 0.212 | 0.174 | 0.102 | 0.417 |
| 2 | chr1_8424984_D | 0.077 | 0.019 | 0.006 | 0.145 | 0.124 | 0.026 | 0.143 | 0.025 | 0.080 | 0.012 | 0.023 | 0.305 | 0.316 | 0.190 | 0.552 | 0.360 | 0.206 | 0.138 | 0.088 | 0.205 |
| 3 | rs1498232 | 0.435 | 0.180 | 0.019 | 1.496 | 1.371 | 1.628 | 0.110 | 0.645 | 0.000 | 0.628 | 0.024 | 0.837 | 0.677 | 0.203 | 0.440 | 0.200 | 0.112 | 0.556 | 0.502 | 0.115 |
| 4 | rs11210892 | 0.349 | 0.085 | 0.130 | 0.533 | 0.562 | 0.035 | 0.081 | 0.082 | 0.250 | 0.008 | 0.405 | 0.278 | 0.344 | 0.230 | 0.492 | 0.018 | 0.090 | 1.126 | 0.051 | 0.300 |
| 5 | rs12129573 | 0.055 | 0.145 | 0.354 | 0.123 | 0.045 | 0.344 | 0.025 | 0.061 | 0.005 | 0.182 | 0.212 | 0.262 | 0.165 | 0.285 | 0.180 | 0.517 | 0.206 | 0.397 | 0.369 | 0.414 |
| 6 | rs1702294 | 0.172 | 0.104 | 0.044 | 0.114 | 0.038 | 0.202 | 0.092 | 2.110 | 0.001 | 0.954 | 0.596 | 0.221 | 0.443 | 0.254 | 0.093 | 0.299 | 0.597 | 0.777 | 0.300 | 0.247 |
| 7 | rs6670165 | 0.110 | 0.317 | 0.026 | 0.169 | 0.071 | 0.334 | 0.045 | 0.226 | 0.040 | 0.554 | 0.043 | 0.119 | 0.081 | 0.055 | 0.063 | 0.002 | 0.569 | 0.034 | 0.297 | 0.145 |
| 8 | rs7523273 | 0.015 | 0.023 | 0.028 | 0.051 | 0.216 | 0.003 | 0.487 | 1.239 | 0.048 | 0.229 | 0.215 | 0.138 | 0.161 | 0.933 | 0.960 | 0.001 | 1.209 | 1.877 | 0.503 | 0.447 |
| 9 | rs10803138 | 0.060 | 0.381 | 0.031 | 0.206 | 0.501 | 0.373 | 1.929 | 1.328 | 1.739 | 0.090 | 0.180 | 0.392 | 0.801 | 0.112 | 0.122 | 0.019 | 0.043 | 0.111 | 0.203 | 0.649 |
| 10 | rs14403 | 0.074 | 0.140 | 0.207 | 0.610 | 0.519 | 0.059 | 0.063 | 0.132 | 0.039 | 0.328 | 0.235 | 0.989 | 0.978 | 0.126 | 0.157 | 0.715 | 0.064 | 0.126 | 0.185 | 0.057 |
| 11 | chr1_243881945_I | 0.142 | 0.059 | 0.882 | 0.158 | 0.230 | 0.264 | 0.009 | 0.030 | 0.299 | 0.684 | 0.123 | 0.060 | 0.060 | 0.077 | 0.271 | 0.462 | 0.068 | 0.028 | 0.184 | 0.150 |
| 12 | rs11682175 | 0.082 | 0.278 | 0.006 | 0.392 | 0.114 | 0.213 | 0.646 | 0.285 | 0.204 | 0.338 | 0.219 | 0.302 | 0.211 | 0.257 | 0.173 | 0.321 | 0.386 | 0.035 | 0.757 | 0.235 |
| 13 | chr2_146436222_I | 0.027 | 0.026 | 0.174 | 0.129 | 0.079 | 0.404 | 0.115 | 0.161 | 0.760 | 0.001 | 0.057 | 0.407 | 0.347 | 0.046 | 0.468 | 0.018 | 0.729 | 0.390 | 0.041 | 0.358 |
| 14 | rs2909457 | 0.413 | 0.146 | 0.018 | 0.066 | 0.059 | 0.023 | 1.311 | 0.466 | 0.004 | 0.050 | 0.011 | 0.156 | 0.396 | 1.206 | 0.946 | 0.082 | 0.179 | 0.748 | 0.336 | 0.935 |
| 15 | rs11693094 | 0.976 | 0.453 | 0.519 | 0.143 | 0.277 | 0.333 | 0.429 | 0.056 | 0.195 | 0.004 | 0.026 | 1.593 | 1.593 | 0.268 | 0.277 | 0.329 | 2.129 | 0.966 | 2.062 | 0.821 |
| 16 | rs59979824 | 0.239 | 0.293 | 0.112 | 1.213 | 0.143 | 0.170 | 0.025 | 0.048 | 0.219 | 0.059 | 0.077 | 0.187 | 0.164 | 0.051 | 0.106 | 0.008 | 0.115 | 0.231 | 0.081 | 0.471 |
| 17 | rs6434928 | 0.501 | 0.677 | 0.356 | 1.117 | 1.036 | 0.494 | 0.029 | 0.011 | 0.756 | 0.088 | 0.169 | 0.328 | 0.304 | 0.649 | 0.921 | 0.088 | 0.266 | 0.120 | 0.480 | 0.239 |
| 18 | rs6704641 | 0.133 | 0.176 | 0.007 | 0.840 | 0.324 | 0.726 | 0.197 | 0.212 | 0.483 | 0.004 | 0.023 | 0.601 | 0.881 | 1.131 | 0.734 | 0.114 | 0.187 | 0.148 | 0.351 | 0.074 |
| 19 | chr2_200825237_I | 0.543 | 0.315 | 0.002 | 0.240 | 0.326 | 0.002 | 0.141 | 0.270 | 0.054 | 0.001 | 0.411 | 0.128 | 0.120 | 0.065 | 0.054 | 0.530 | 0.250 | 0.033 | 0.428 | 0.064 |
| 20 | rs11685299 | 0.580 | 1.008 | 0.493 | 0.221 | 0.164 | 0.086 | 0.103 | 0.608 | 0.287 | 0.412 | 0.547 | 0.096 | 0.173 | 0.848 | 0.234 | 0.066 | 0.871 | 0.509 | 1.040 | 0.618 |
| 21 | rs6704768 | 0.030 | 0.201 | 0.070 | 0.022 | 0.090 | 0.028 | 0.119 | 0.138 | 0.563 | 0.055 | 0.235 | 0.076 | 0.148 | 0.153 | 0.725 | 0.040 | 0.435 | 0.034 | 1.076 | 0.168 |
| 22 | rs17194490 | 0.036 | 0.061 | 0.001 | 0.166 | 0.538 | 0.005 | 0.154 | 0.160 | 0.016 | 0.082 | 0.034 | 0.106 | 0.091 | 0.426 | 0.977 | 0.007 | 0.097 | 0.123 | 0.122 | 0.107 |
| 23 | rs4330281 | 0.888 | 0.188 | 0.078 | 0.434 | 0.256 | 0.035 | 0.601 | 1.605 | 0.047 | 0.324 | 0.344 | 0.233 | 0.119 | 0.097 | 0.158 | 0.007 | 0.033 | 0.429 | 0.094 | 0.288 |
| 24 | rs75968099 | 0.693 | 1.279 | 0.447 | 0.009 | 0.007 | 0.354 | 0.142 | 1.141 | 0.066 | 0.317 | 0.155 | 0.122 | 0.073 | 0.096 | 0.532 | 1.374 | 0.040 | 0.145 | 2.179 | 0.850 |
| 25 | rs2535627 | 1.277 | 0.826 | 0.230 | 0.786 | 0.700 | 0.130 | 0.805 | 1.063 | 0.281 | 0.769 | 0.689 | 3.133 | 1.650 | 1.435 | 2.322 | 0.117 | 0.089 | 0.235 | 0.134 | 0.392 |
| 26 | rs832187 | 0.818 | 0.075 | 0.014 | 0.215 | 0.074 | 0.007 | 0.695 | 0.026 | 0.125 | 0.096 | 0.374 | 0.217 | 0.142 | 0.181 | 0.428 | 1.323 | 0.661 | 0.263 | 0.078 | 0.041 |
| 27 | rs7432375 | 0.102 | 0.134 | 0.004 | 0.129 | 0.176 | 0.733 | 0.264 | 0.027 | 0.189 | 0.143 | 0.131 | 0.175 | 0.097 | 0.061 | 0.172 | 0.049 | 0.208 | 0.601 | 0.221 | 0.338 |
| 28 | chr3_180594593_I | 0.136 | 0.049 | 0.012 | 0.577 | 0.574 | 0.707 | 0.037 | 0.037 | 0.432 | 0.277 | 0.282 | 0.530 | 0.263 | 0.447 | 0.274 | 0.450 | 0.105 | 0.664 | 0.202 | 0.140 |
| 29 | rs9841616 | 0.070 | 0.034 | 0.094 | 1.102 | 1.857 | 2.693 | 0.871 | 0.199 | 0.053 | 0.112 | 1.724 | 0.967 | 0.344 | 0.407 | 0.262 | 1.155 | 0.420 | 0.055 | 1.653 | 0.267 |
| 30 | rs215411 | 0.037 | 0.108 | 0.035 | 0.036 | 0.484 | 0.085 | 0.529 | 0.524 | 0.482 | 0.076 | 1.152 | 0.280 | 0.227 | 0.198 | 0.062 | 0.010 | 0.202 | 0.055 | 0.128 | 0.427 |
| 31 | rs10520163 | 0.170 | 0.024 | 0.064 | 0.086 | 0.277 | 0.322 | 0.089 | 0.375 | 0.790 | 0.004 | 0.063 | 0.221 | 0.203 | 0.096 | 0.090 | 0.247 | 0.107 | 0.036 | 0.260 | 0.139 |
| 32 | rs1106568 | 0.033 | 0.043 | 0.640 | 0.184 | 0.630 | 0.216 | 0.401 | 0.994 | 0.001 | 0.017 | 0.005 | 0.102 | 0.111 | 0.117 | 0.111 | 0.114 | 0.239 | 0.177 | 0.289 | 0.242 |
| 33 | rs1501357 | 0.128 | 0.897 | 0.349 | 0.227 | 1.003 | 0.056 | 0.157 | 0.133 | 0.005 | 0.178 | 0.037 | 0.334 | 0.881 | 0.092 | 0.148 | 0.485 | 0.862 | 1.511 | 0.623 | 1.108 |
| 34 | rs4391122 | 0.356 | 0.225 | 0.027 | 0.343 | 0.704 | 0.554 | 1.861 | 0.709 | 0.775 | 0.052 | 0.005 | 0.384 | 0.196 | 1.302 | 0.740 | 0.017 | 0.208 | 0.032 | 0.564 | 0.162 |
| 35 | rs16867576 | 0.398 | 0.612 | 0.566 | 0.061 | 0.080 | 0.013 | 1.105 | 0.243 | 0.039 | 0.532 | 0.685 | 0.372 | 0.116 | 0.282 | 0.129 | 0.844 | 0.219 | 0.233 | 0.055 | 0.350 |
| 36 | rs4388249 | 0.036 | 0.131 | 0.790 | 0.396 | 0.280 | 0.005 | 0.860 | 0.474 | 0.046 | 0.150 | 0.316 | 0.171 | 0.212 | 0.171 | 0.478 | 0.321 | 0.256 | 0.049 | 1.054 | 1.202 |
| 37 | rs10043984 | 0.021 | 0.338 | 0.090 | 0.090 | 0.585 | 1.008 | 0.348 | 0.055 | 0.176 | 0.036 | 0.024 | 0.255 | 0.119 | 0.159 | 0.212 | 1.668 | 0.102 | 0.500 | 0.231 | 0.319 |
| 38 | rs3849046 | 0.061 | 0.234 | 0.087 | 0.751 | 0.600 | 0.133 | 0.026 | 0.059 | 0.028 | 0.557 | 0.296 | 0.703 | 0.701 | 0.370 | 0.277 | 0.213 | 0.057 | 0.789 | 0.464 | 0.408 |
| 39 | chr5_140143664_I | 0.129 | 0.046 | 0.014 | 0.389 | 1.054 | 0.269 | 0.944 | 1.329 | 0.427 | 0.195 | 0.966 | 0.129 | 0.318 | 0.278 | 0.742 | 0.046 | 0.267 | 0.042 | 0.452 | 0.167 |
| 40 | rs111294930 | 0.095 | 0.866 | 0.017 | 0.625 | 0.696 | 0.923 | 0.044 | 0.212 | 0.323 | 0.250 | 0.075 | 0.418 | 0.512 | 0.054 | 0.069 | 0.016 | 0.512 | 0.090 | 0.236 | 0.770 |
| 41 | rs2973155 | 0.035 | 0.097 | 0.045 | 0.773 | 1.777 | 0.843 | 0.077 | 0.186 | 0.095 | 0.169 | 0.026 | 0.139 | 0.176 | 0.069 | 0.067 | 0.001 | 0.770 | 0.144 | 0.033 | 0.187 |
| 42 | rs12522290 | 0.153 | 0.188 | 0.645 | 0.334 | 1.292 | 0.017 | 0.006 | 0.503 | 0.218 | 0.373 | 0.403 | 0.381 | 0.308 | 0.138 | 0.262 | 0.023 | 0.873 | 0.711 | 1.094 | 1.293 |
| 43 | rs11740474 | 0.583 | 1.340 | 0.241 | 0.278 | 0.687 | 0.050 | 0.101 | 0.048 | 0.014 | 0.000 | 0.008 | 0.182 | 0.391 | 0.274 | 0.645 | 0.025 | 0.274 | 0.037 | 0.560 | 0.554 |
| 44 | rs1339227 | 0.073 | 0.199 | 0.056 | 0.009 | 0.065 | 0.321 | 0.359 | 0.379 | 0.584 | 0.015 | 0.006 | 0.131 | 0.150 | 0.061 | 0.182 | 0.001 | 0.409 | 1.421 | 2.901 | 0.496 |
| 45 | chr6_84280274_D | 0.478 | 1.376 | 0.239 | 0.362 | 0.382 | 0.021 | 0.097 | 0.070 | 0.543 | 0.014 | 0.019 | 0.094 | 0.082 | 1.077 | 0.938 | 1.608 | 0.094 | 0.097 | 0.233 | 0.116 |
| 46 | chr7_2025096_I | 0.463 | 0.234 | 0.900 | 0.044 | 0.056 | 0.071 | 0.662 | 0.120 | 0.064 | 0.208 | 0.371 | 0.651 | 0.679 | 0.292 | 0.263 | 0.552 | 0.051 | 0.692 | 0.453 | 1.381 |
| 47 | rs12704290 | 1.064 | 1.461 | 0.179 | 0.482 | 0.512 | 0.325 | 0.104 | 0.439 | 0.337 | 0.211 | 0.780 | 0.337 | 0.300 | 0.476 | 0.169 | 0.022 | 0.547 | 0.260 | 1.407 | 0.677 |
| 48 | rs6466055 | 1.019 | 0.566 | 0.003 | 0.087 | 0.472 | 0.167 | 0.025 | 0.153 | 0.653 | 0.020 | 0.537 | 0.335 | 0.286 | 0.226 | 0.086 | 0.306 | 0.143 | 0.234 | 0.462 | 0.364 |
| 49 | rs211829 | 0.503 | 0.141 | 0.180 | 0.487 | 0.072 | 0.009 | 0.038 | 0.311 | 0.062 | 0.146 | 0.530 | 0.452 | 0.472 | 0.487 | 0.243 | 0.001 | 0.166 | 0.036 | 0.654 | 0.613 |
| 50 | rs13240464 | 0.067 | 0.893 | 0.217 | 0.703 | 0.740 | 0.368 | 0.208 | 0.085 | 0.111 | 0.088 | 0.034 | 0.657 | 0.537 | 0.345 | 0.335 | 0.009 | 0.250 | 0.124 | 0.016 | 0.046 |
| 51 | rs3735025 | 0.201 | 0.381 | 0.462 | 0.331 | 1.381 | 2.761 | 0.161 | 0.262 | 0.027 | 0.071 | 0.158 | 0.199 | 0.225 | 0.344 | 0.698 | 1.216 | 0.075 | 0.133 | 0.134 | 0.407 |
| 52 | rs10503253 | 1.092 | 0.595 | 1.227 | 0.152 | 0.065 | 0.218 | 0.228 | 0.518 | 0.196 | 0.458 | 0.113 | 0.087 | 0.068 | 0.833 | 0.874 | 0.821 | 0.101 | 0.231 | 0.582 | 0.198 |
| 53 | rs6984242 | 0.203 | 0.455 | 0.409 | 0.729 | 0.818 | 0.019 | 2.960 | 2.028 | 0.148 | 0.164 | 0.063 | 0.157 | 0.083 | 0.138 | 0.507 | 0.005 | 0.167 | 0.634 | 0.066 | 0.607 |
| 54 | rs7819570 | 0.272 | 0.523 | 0.027 | 0.142 | 0.079 | 0.019 | 0.217 | 0.429 | 2.345 | 0.083 | 0.010 | 0.158 | 0.189 | 0.284 | 0.797 | 0.000 | 0.276 | 0.053 | 0.288 | 0.281 |
| 55 | rs36068923 | 0.108 | 0.061 | 0.043 | 0.186 | 0.013 | 0.198 | 0.375 | 0.106 | 0.204 | 0.174 | 0.080 | 0.295 | 0.185 | 0.090 | 0.325 | 0.006 | 0.140 | 0.217 | 0.086 | 0.636 |
| 56 | rs4129585 | 0.949 | 1.124 | 0.389 | 0.557 | 0.385 | 0.045 | 0.424 | 0.256 | 0.219 | 0.285 | 0.062 | 0.035 | 0.103 | 0.468 | 0.637 | 0.366 | 0.038 | 0.122 | 0.116 | 0.174 |
| 57 | rs11139497 | 0.076 | 0.094 | 0.194 | 0.138 | 0.265 | 0.002 | 0.488 | 0.038 | 0.050 | 0.033 | 0.349 | 0.120 | 0.185 | 0.376 | 0.100 | 0.629 | 0.408 | 1.173 | 0.778 | 1.894 |
| 58 | rs11191419 | 1.141 | 1.372 | 0.393 | 0.498 | 0.519 | 0.492 | 0.100 | 0.056 | 0.002 | 0.117 | 0.000 | 0.231 | 0.478 | 0.133 | 0.070 | 0.172 | 1.311 | 0.367 | 0.466 | 0.705 |
| 59 | rs55833108 | 0.055 | 0.108 | 0.022 | 0.582 | 0.545 | 0.458 | 0.207 | 0.216 | 0.007 | 1.909 | 0.273 | 0.473 | 0.578 | 0.034 | 0.667 | 0.246 | 0.672 | 0.662 | 0.404 | 1.258 |
| 60 | rs11027857 | 0.011 | 0.152 | 0.039 | 0.050 | 0.152 | 0.479 | 0.948 | 0.662 | 0.191 | 0.020 | 0.048 | 0.188 | 0.127 | 0.184 | 0.077 | 0.002 | 0.026 | 0.414 | 0.732 | 0.266 |
| 61 | chr11_46350213_D | 1.095 | 0.151 | 0.201 | 0.315 | 0.078 | 0.003 | 0.270 | 0.079 | 0.066 | 0.016 | 0.004 | 0.166 | 0.256 | 0.086 | 0.091 | 0.009 | 0.428 | 0.009 | 0.180 | 0.097 |
| 62 | rs9420 | 0.024 | 0.263 | 0.122 | 0.069 | 0.016 | 0.458 | 0.037 | 0.619 | 0.078 | 0.064 | 0.037 | 1.105 | 0.960 | 0.714 | 0.249 | 0.057 | 0.201 | 0.285 | 0.618 | 0.259 |
| 63 | rs12421382 | 0.116 | 0.462 | 0.052 | 1.588 | 0.346 | 0.004 | 0.195 | 0.083 | 0.016 | 0.074 | 0.007 | 0.094 | 0.094 | 0.727 | 0.172 | 0.376 | 0.436 | 0.923 | 0.126 | 0.427 |
| 64 | rs2514218 | 0.024 | 0.013 | 0.025 | 0.008 | 0.017 | 0.088 | 0.075 | 0.109 | 0.823 | 0.005 | 0.475 | 0.123 | 0.114 | 0.028 | 0.045 | 0.002 | 0.125 | 0.365 | 0.537 | 0.467 |
| 65 | rs77502336 | 0.315 | 0.109 | 0.028 | 0.047 | 0.569 | 0.227 | 0.874 | 0.428 | 0.421 | 0.056 | 0.443 | 0.569 | 0.558 | 0.186 | 0.279 | 1.035 | 0.160 | 0.929 | 0.064 | 0.847 |
| 66 | rs55661361 | 0.050 | 0.603 | 0.753 | 1.203 | 1.711 | 0.328 | 0.018 | 0.062 | 0.085 | 0.010 | 0.039 | 1.376 | 1.785 | 0.101 | 0.052 | 0.010 | 0.127 | 0.958 | 0.370 | 0.327 |
| 67 | rs10791097 | 0.017 | 0.135 | 0.001 | 0.016 | 0.098 | 0.417 | 0.283 | 0.252 | 0.010 | 0.010 | 0.025 | 0.282 | 0.132 | 0.637 | 0.271 | 0.004 | 0.070 | 0.074 | 0.218 | 0.069 |
| 68 | rs75059851 | 0.019 | 0.216 | 0.052 | 0.364 | 0.902 | 0.053 | 0.229 | 0.152 | 0.137 | 0.379 | 0.162 | 0.218 | 0.624 | 0.407 | 0.197 | 0.224 | 0.217 | 0.095 | 0.064 | 0.022 |
| 69 | rs2007044 | 0.015 | 0.019 | 0.020 | 0.542 | 0.551 | 1.056 | 0.944 | 2.328 | 0.388 | 0.139 | 0.025 | 2.158 | 2.266 | 0.726 | 0.354 | 0.131 | 0.391 | 0.305 | 0.768 | 0.623 |
| 70 | rs2239063 | 0.017 | 0.036 | 0.001 | 0.070 | 0.006 | 0.076 | 0.024 | 0.042 | 0.001 | 0.684 | 0.123 | 0.198 | 0.162 | 0.084 | 0.299 | 0.499 | 0.636 | 0.222 | 0.074 | 0.397 |
| 71 | rs679087 | 0.018 | 0.097 | 0.077 | 0.325 | 0.027 | 0.004 | 0.527 | 0.101 | 0.029 | 0.009 | 0.152 | 0.425 | 0.761 | 0.254 | 0.278 | 0.001 | 0.049 | 0.101 | 0.302 | 0.129 |
| 72 | rs324017 | 0.092 | 0.378 | 0.223 | 0.160 | 0.063 | 0.005 | 0.302 | 0.219 | 0.013 | 0.015 | 0.005 | 0.129 | 0.134 | 0.266 | 0.071 | 0.089 | 0.238 | 0.257 | 0.091 | 0.720 |
| 73 | rs4240748 | 0.260 | 0.669 | 0.003 | 0.068 | 0.450 | 0.045 | 0.237 | 0.837 | 0.346 | 0.023 | 0.160 | 0.430 | 0.309 | 0.043 | 0.048 | 0.218 | 0.620 | 0.079 | 1.015 | 0.060 |
| 74 | rs10860964 | 0.592 | 0.223 | 0.033 | 0.587 | 0.191 | 0.005 | 0.407 | 0.532 | 0.441 | 0.013 | 0.003 | 0.238 | 0.226 | 0.139 | 0.202 | 0.018 | 0.119 | 0.401 | 0.418 | 0.029 |
| 75 | rs4766428 | 0.111 | 0.180 | 0.007 | 0.040 | 0.041 | 0.477 | 0.103 | 0.232 | 0.263 | 0.112 | 0.629 | 0.238 | 0.328 | 0.136 | 0.109 | 0.020 | 0.565 | 0.007 | 0.847 | 0.098 |
| 76 | rs2851447 | 0.062 | 0.316 | 0.048 | 0.073 | 0.637 | 0.059 | 0.019 | 0.055 | 0.594 | 0.115 | 0.239 | 1.162 | 0.685 | 0.500 | 0.518 | 0.454 | 0.040 | 0.134 | 0.050 | 0.468 |
| 77 | rs2068012 | 0.063 | 0.186 | 0.047 | 0.033 | 0.094 | 0.038 | 0.568 | 1.281 | 1.011 | 1.455 | 2.790 | 1.108 | 0.606 | 0.037 | 0.123 | 0.148 | 0.177 | 0.797 | 0.124 | 0.907 |
| 78 | rs2332700 | 0.165 | 1.036 | 0.332 | 0.084 | 0.289 | 0.092 | 0.180 | 0.025 | 0.044 | 0.062 | 0.171 | 0.131 | 0.110 | 0.413 | 0.278 | 0.870 | 0.049 | 0.036 | 0.142 | 0.110 |
| 79 | rs2693698 | 0.064 | 0.031 | 0.206 | 0.133 | 0.512 | 0.004 | 0.815 | 0.425 | 0.767 | 0.148 | 0.025 | 1.081 | 1.004 | 0.383 | 0.288 | 0.161 | 1.502 | 1.233 | 0.257 | 0.454 |
| 80 | rs12887734 | 0.063 | 0.015 | 0.002 | 0.124 | 0.011 | 0.185 | 0.344 | 0.479 | 0.180 | 0.194 | 0.688 | 0.197 | 0.195 | 0.103 | 0.151 | 0.079 | 0.238 | 0.405 | 0.318 | 0.087 |
| 81 | rs56205728 | 0.038 | 0.091 | 0.004 | 0.143 | 0.019 | 0.031 | 0.141 | 0.041 | 0.971 | 0.891 | 0.123 | 1.855 | 1.273 | 0.109 | 0.191 | 0.311 | 1.854 | 0.484 | 0.198 | 0.284 |
| 82 | rs12903146 | 0.066 | 0.067 | 0.158 | 0.033 | 0.165 | 0.024 | 0.891 | 0.780 | 0.003 | 0.179 | 0.209 | 0.047 | 0.052 | 0.349 | 0.120 | 0.533 | 0.176 | 0.103 | 0.843 | 0.156 |
| 83 | rs12148337 | 0.041 | 0.162 | 0.037 | 0.075 | 0.094 | 0.511 | 0.395 | 0.543 | 0.243 | 0.248 | 0.083 | 0.071 | 0.067 | 0.119 | 0.370 | 0.011 | 0.266 | 0.239 | 1.307 | 0.329 |
| 84 | rs190065944 | 0.026 | 0.029 | 0.037 | 0.011 | 0.038 | 0.157 | 0.112 | 0.045 | 1.542 | 0.419 | 1.424 | 0.618 | 0.788 | 0.508 | 0.343 | 0.007 | 0.021 | 0.364 | 0.138 | 0.050 |
| 85 | rs8042374 | 0.852 | 0.495 | 0.218 | 0.034 | 0.126 | 0.060 | 0.036 | 0.020 | 0.018 | 0.246 | 0.482 | 0.671 | 0.442 | 0.792 | 1.463 | 0.008 | 0.063 | 0.068 | 0.384 | 0.681 |
| 86 | rs950169 | 0.370 | 0.402 | 0.004 | 0.205 | 0.047 | 0.006 | 0.334 | 0.480 | 0.063 | 0.312 | 0.246 | 0.275 | 0.091 | 0.192 | 0.124 | 0.869 | 0.118 | 0.216 | 0.105 | 0.500 |
| 87 | rs4702 | 0.008 | 0.004 | 0.058 | 0.031 | 0.964 | 0.038 | 0.546 | 0.977 | 0.087 | 0.061 | 0.258 | 0.610 | 0.419 | 0.270 | 1.039 | 0.394 | 0.069 | 0.041 | 0.431 | 0.171 |
| 88 | rs9922678 | 0.145 | 0.065 | 0.392 | 0.053 | 0.088 | 0.025 | 0.014 | 0.067 | 0.005 | 0.158 | 0.010 | 0.285 | 0.232 | 0.203 | 0.402 | 0.146 | 0.088 | 0.130 | 1.432 | 0.530 |
| 89 | rs7405404 | 0.545 | 0.254 | 0.019 | 0.093 | 0.743 | 0.607 | 0.044 | 0.452 | 0.171 | 0.106 | 0.342 | 0.167 | 0.170 | 0.266 | 0.178 | 0.016 | 0.012 | 0.377 | 2.190 | 0.300 |
| 90 | rs12691307 | 0.278 | 0.972 | 0.452 | 0.224 | 0.018 | 0.128 | 1.261 | 0.894 | 0.164 | 0.112 | 0.178 | 0.208 | 0.327 | 0.064 | 0.159 | 0.778 | 0.018 | 0.043 | 0.213 | 0.280 |
| 91 | rs8044995 | 0.163 | 0.262 | 0.057 | 0.329 | 0.122 | 0.032 | 0.287 | 0.407 | 0.063 | 0.001 | 0.371 | 0.125 | 0.197 | 0.220 | 0.301 | 0.002 | 0.371 | 0.014 | 0.140 | 0.240 |
| 92 | rs4523957 | 0.038 | 0.110 | 0.269 | 0.106 | 1.177 | 0.058 | 1.432 | 1.111 | 0.628 | 0.008 | 0.051 | 0.296 | 0.187 | 0.052 | 0.267 | 0.014 | 0.320 | 0.027 | 0.241 | 0.082 |
| 93 | rs8082590 | 0.039 | 0.198 | 0.377 | 0.038 | 0.030 | 0.063 | 0.032 | 0.349 | 0.431 | 0.532 | 0.252 | 0.347 | 0.115 | 2.243 | 2.540 | 0.036 | 0.282 | 0.119 | 0.887 | 0.157 |
| 94 | chr18_52749216_D | 0.268 | 0.165 | 0.065 | 0.043 | 0.144 | 1.047 | 0.534 | 0.124 | 0.720 | 0.225 | 0.419 | 0.162 | 0.297 | 1.402 | 0.281 | 0.137 | 0.154 | 0.295 | 0.070 | 0.316 |
| 95 | rs9636107 | 0.826 | 1.659 | 2.099 | 0.128 | 0.081 | 0.030 | 0.616 | 1.866 | 0.392 | 0.003 | 0.018 | 0.602 | 0.378 | 0.218 | 0.251 | 0.103 | 0.101 | 0.218 | 0.414 | 0.225 |
| 96 | rs715170 | 0.157 | 0.048 | 0.068 | 2.101 | 1.499 | 0.229 | 0.815 | 1.068 | 0.548 | 0.041 | 0.025 | 0.081 | 0.077 | 0.042 | 0.133 | 0.210 | 0.117 | 0.145 | 0.086 | 0.124 |
| 97 | rs2905426 | 0.977 | 1.113 | 0.907 | 0.629 | 0.472 | 0.188 | 0.210 | 0.044 | 0.002 | 0.109 | 0.576 | 1.497 | 1.326 | 0.046 | 0.269 | 0.028 | 0.025 | 0.452 | 0.058 | 0.438 |
| 98 | rs2053079 | 0.244 | 0.648 | 0.494 | 0.045 | 0.016 | 0.024 | 0.174 | 0.015 | 0.546 | 0.049 | 0.100 | 1.246 | 1.340 | 0.122 | 0.539 | 0.005 | 0.087 | 0.186 | 0.533 | 0.141 |
| 99 | rs56873913 | 0.424 | 0.880 | 0.473 | 0.082 | 0.110 | 0.045 | 1.196 | 0.247 | 0.148 | 0.137 | 0.427 | 0.722 | 0.300 | 0.054 | 0.071 | 0.232 | 0.093 | 0.940 | 0.062 | 0.107 |
| 100 | rs6065094 | 0.357 | 0.089 | 0.026 | 1.099 | 0.771 | 1.099 | 0.695 | 0.241 | 0.343 | 0.770 | 0.440 | 0.111 | 0.141 | 1.942 | 1.057 | 1.218 | 0.170 | 0.782 | 0.484 | 1.116 |
| 101 | rs7267348 | 0.015 | 0.169 | 0.520 | 0.006 | 0.183 | 0.029 | 0.158 | 0.634 | 0.069 | 0.015 | 0.164 | 0.561 | 0.527 | 0.406 | 0.320 | 2.722 | 0.300 | 0.973 | 0.708 | 1.428 |
| 102 | chr22_39987017_D | 0.257 | 0.074 | 0.006 | 0.443 | 0.174 | 0.066 | 0.930 | 0.694 | 0.013 | 0.031 | 0.008 | 0.751 | 1.245 | 0.151 | 0.378 | 0.453 | 0.686 | 0.052 | 0.151 | 0.128 |
| 103 | rs9607782 | 0.021 | 0.024 | 0.256 | 0.013 | 0.045 | 0.239 | 0.035 | 0.101 | 0.597 | 0.754 | 0.158 | 0.304 | 0.101 | 0.726 | 3.789 | 0.019 | 0.184 | 0.072 | 0.143 | 0.131 |
| 104 | rs1023500 | 0.009 | 0.567 | 0.001 | 0.348 | 0.302 | 0.521 | 0.098 | 0.345 | 0.114 | 0.523 | 0.263 | 1.142 | 1.557 | 0.461 | 0.092 | 0.183 | 0.166 | 0.026 | 0.251 | 0.047 |
| 105 | rs6002655 | 0.086 | 0.040 | 0.044 | 0.063 | 0.245 | 0.084 | 0.041 | 0.317 | 0.011 | 0.095 | 0.090 | 0.341 | 0.630 | 0.070 | 0.685 | 0.846 | 0.336 | 0.152 | 0.982 | 0.121 |

Results of F-tests for each SNP within task and region of interest. Numbers represent -log10 p-value of family wise error correction for respective region of interest (ROI). SNP single nucleotide polymorphism; L left; R right; Hi hippocampus; pgACC perigenual anterior cingulate cortex; DLPFC dorsolateral prefrontal cortex; VStr ventral striatum; AMY amygdala; DMPFC dorsomedial prefrontal cortex; Pcu/PCC precuneus/posterior cingulate cortex; TPJ temporoparietal junction; EM episodic memory; WM working memory; RP reward processing; FMT face matching task; ToM Theory of Mind.

**Supplementary Figure S1:** Subject exclusion criteria


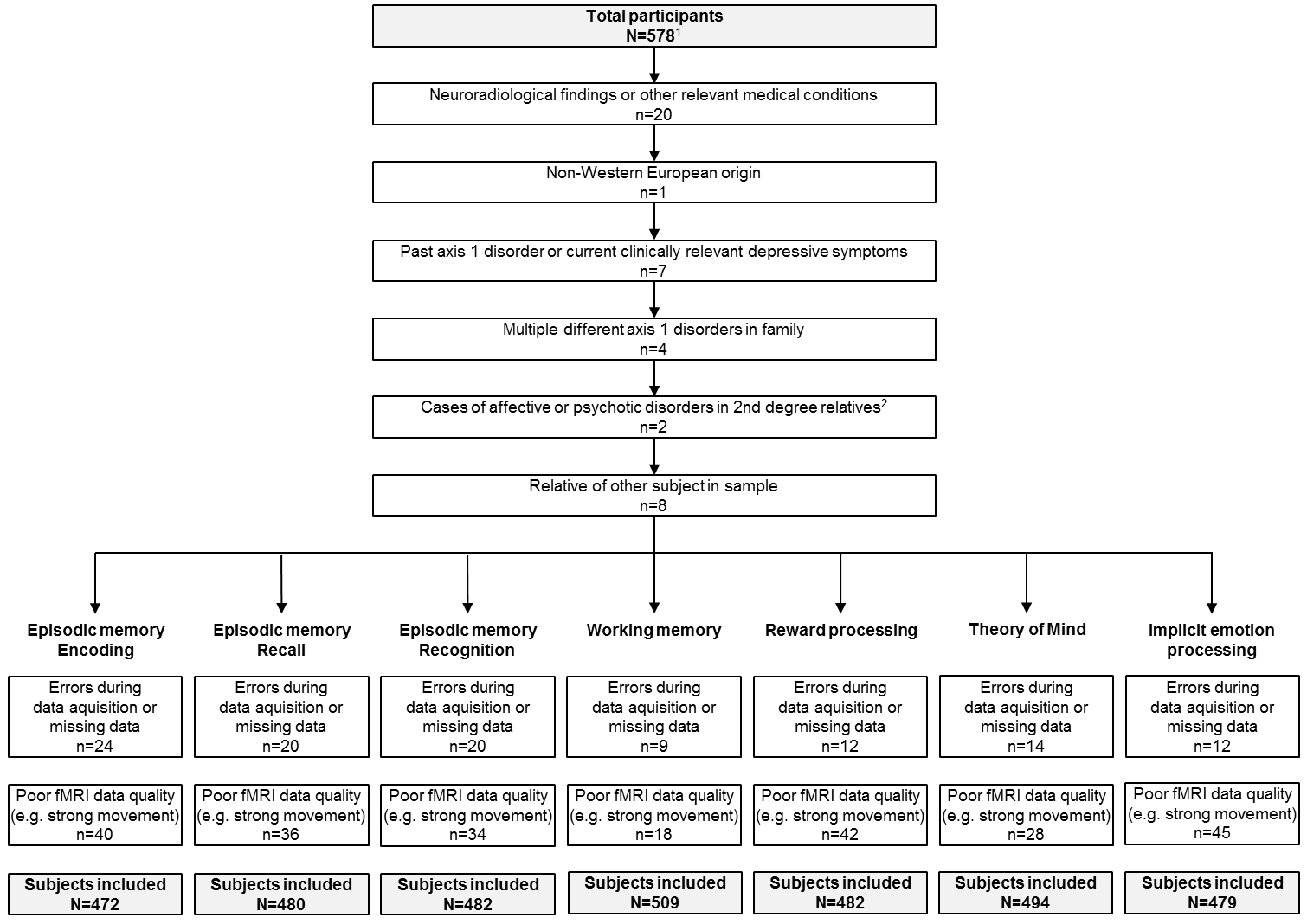


^1^n=333 subjects without familial liability for psychiatric disorders, n=245 unaffected subjects with at least one first-degree relative diagnosed with depression, bipolar disorder or schizophrenia; ^2^criterion pertains to subjects without familial liability for psychiatric disorders only; fMRI functional magnetic resonance imaging

**Supplementary Figure S2:** Regions of interest


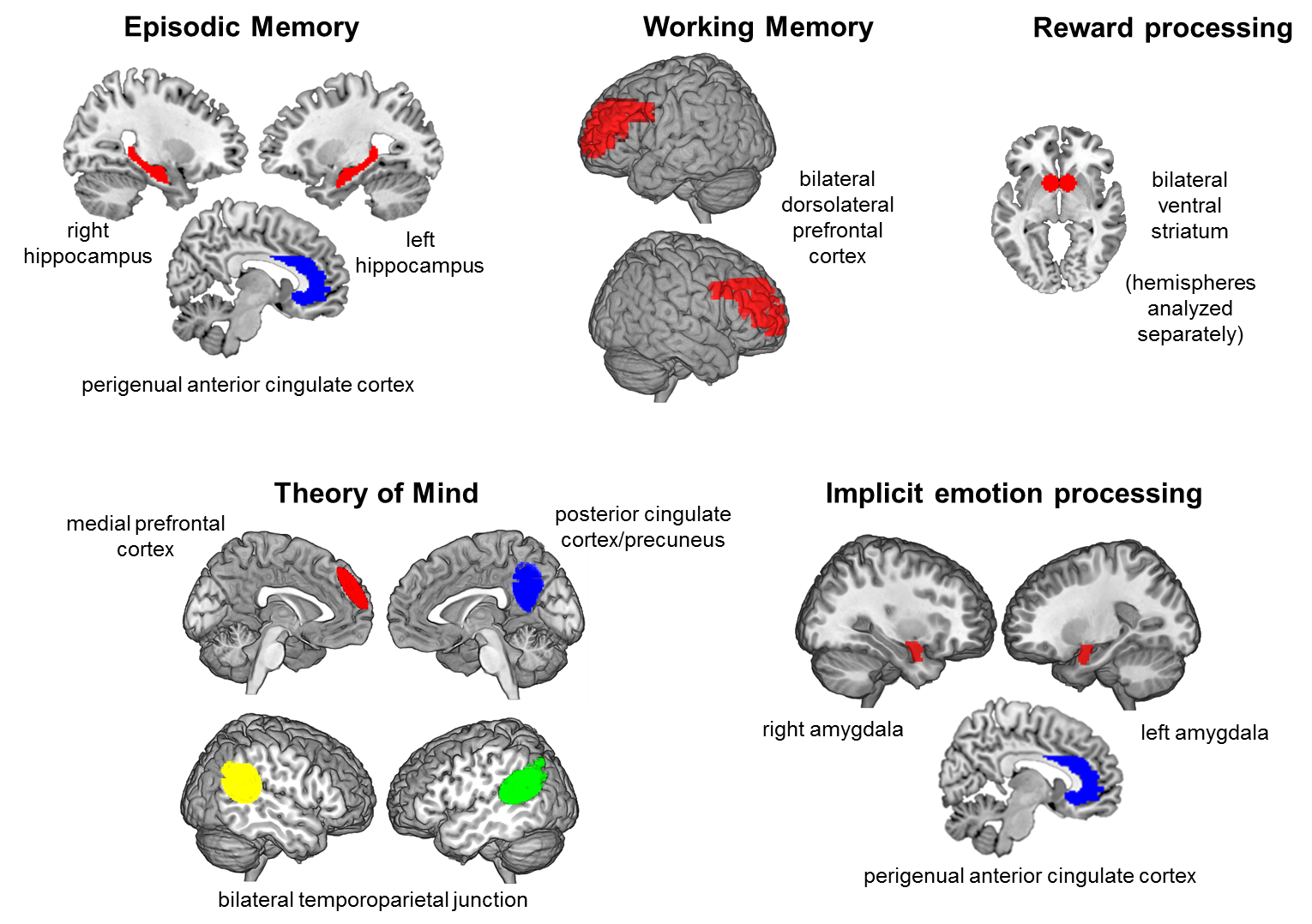


**Supplementary Figure S3:** Effects of SNPs not associated with schizophrenia on regional brain activation

**
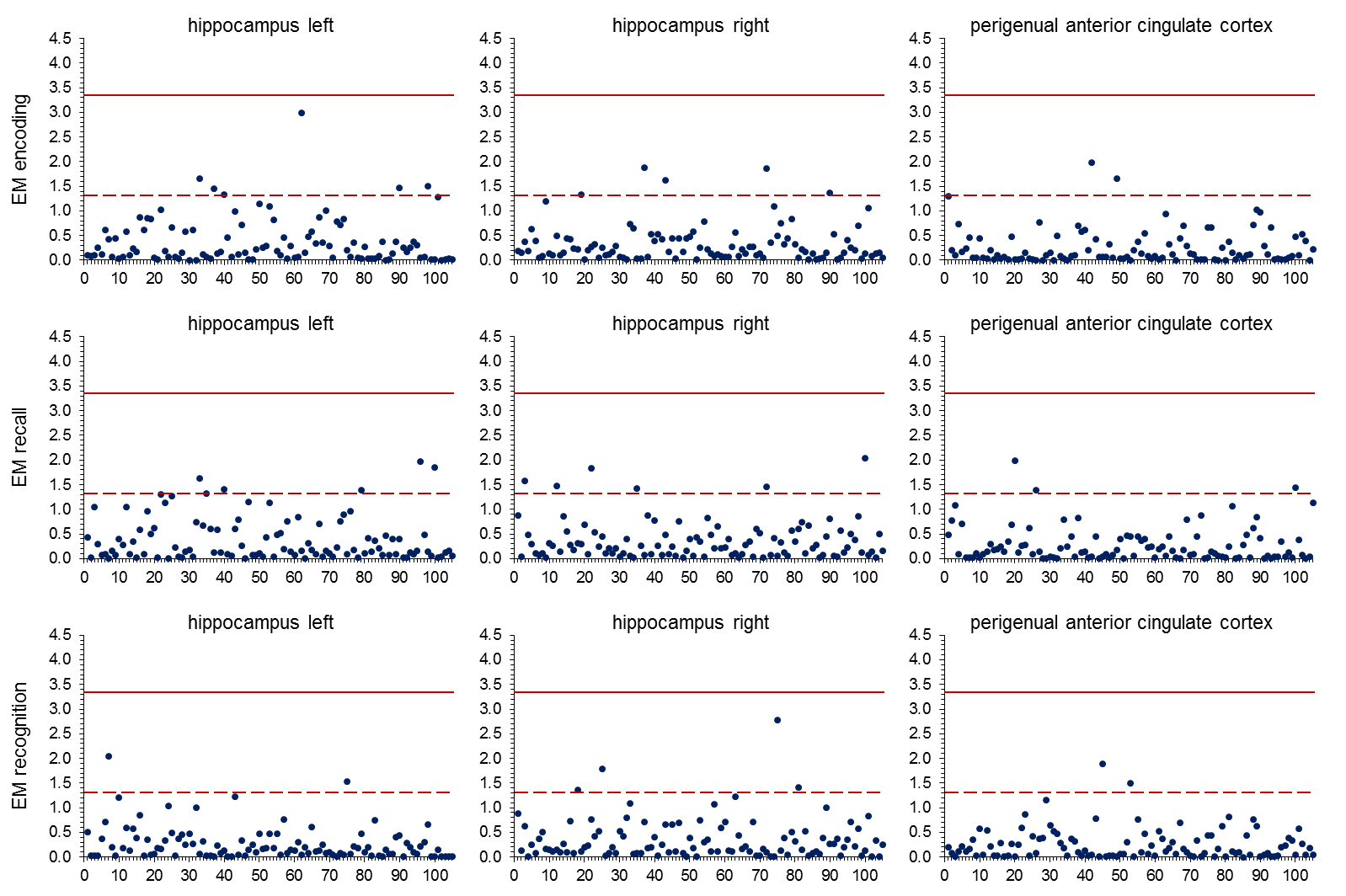
**

**
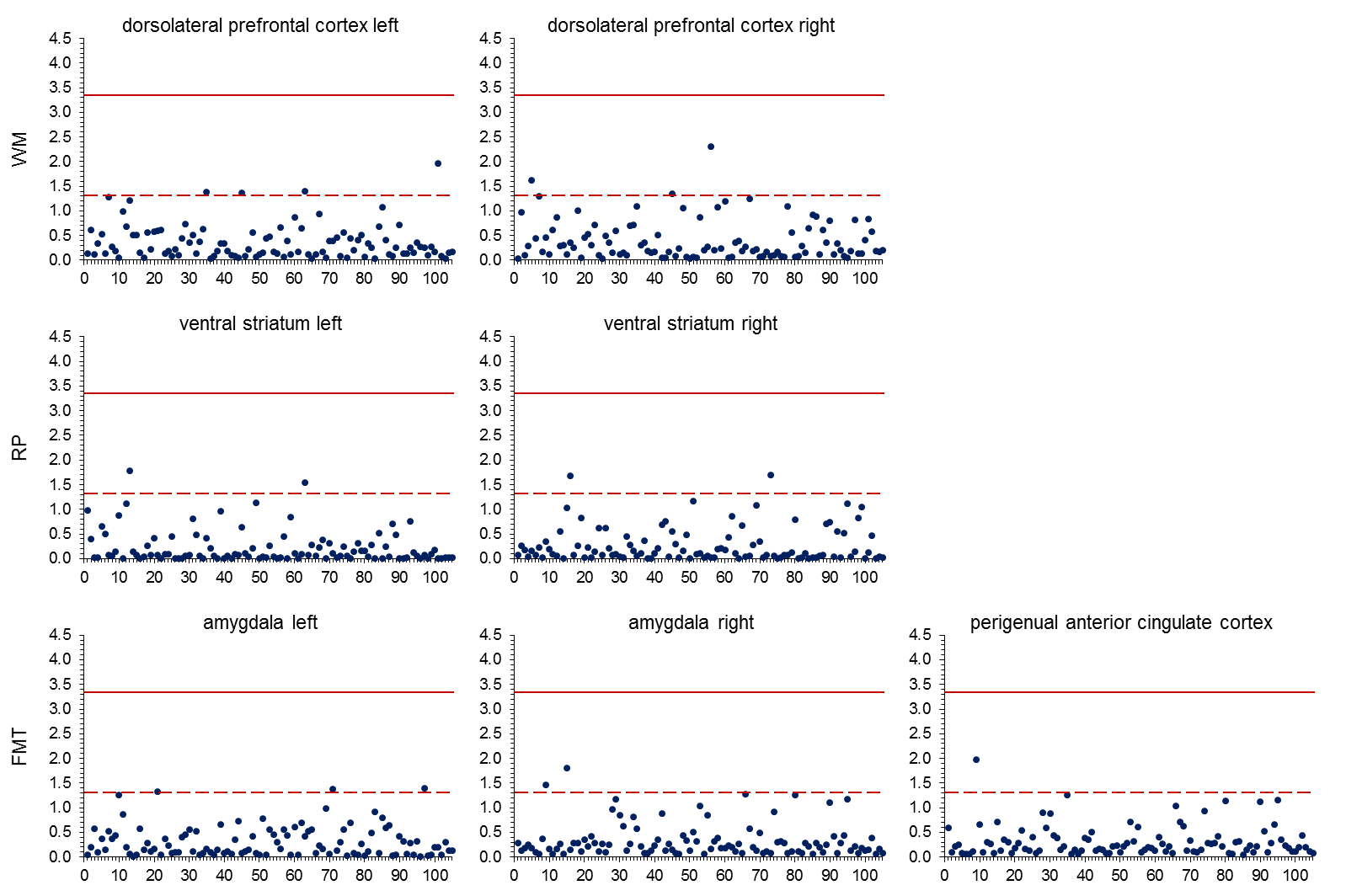
**

**
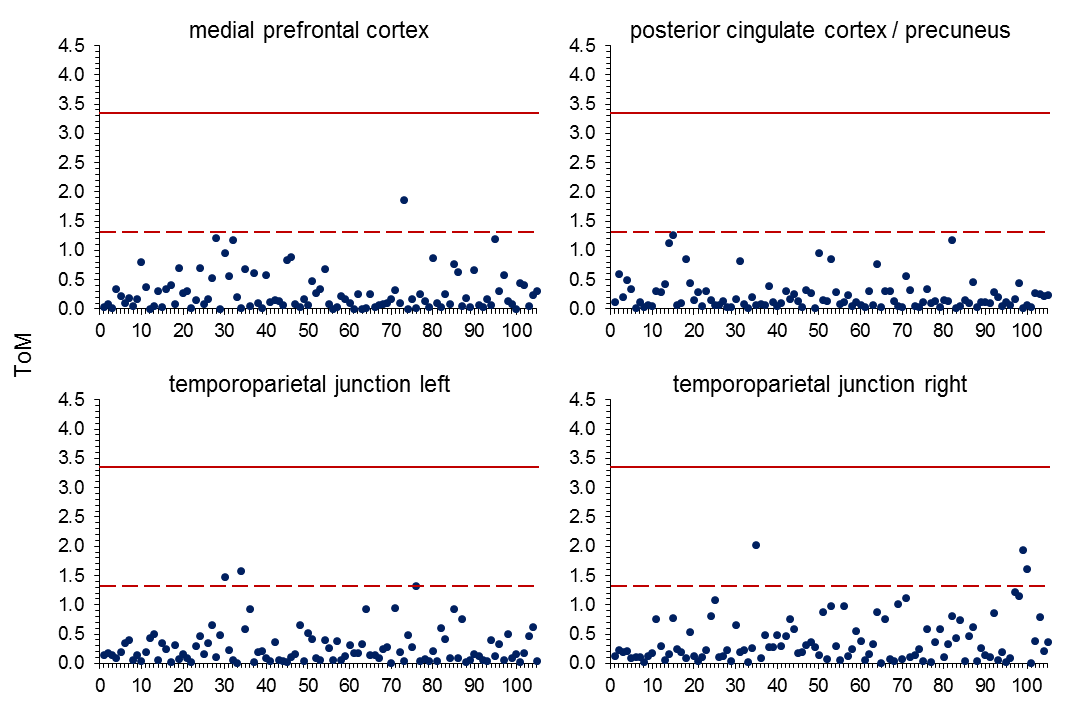
**

Results of analyses of single SNPs not associated with schizophrenia for regions of interest in all tasks. Dashed line indicates –log10 p-value of family wise error correction for respective region of interest. Red line indicates –log10 p-value of correction for multiple tests (p=4x10^-4^). x-axis: number of SNP, ordered by position within chromosome, y-axis: -log10 p-value of F-tests.

EM episodic memory; WM working memory; RP reward processing; FMT face matching task; ToM Theory of Mind.

**Supplementary Figure S4:** Association results of RPS with intermediate phenotype


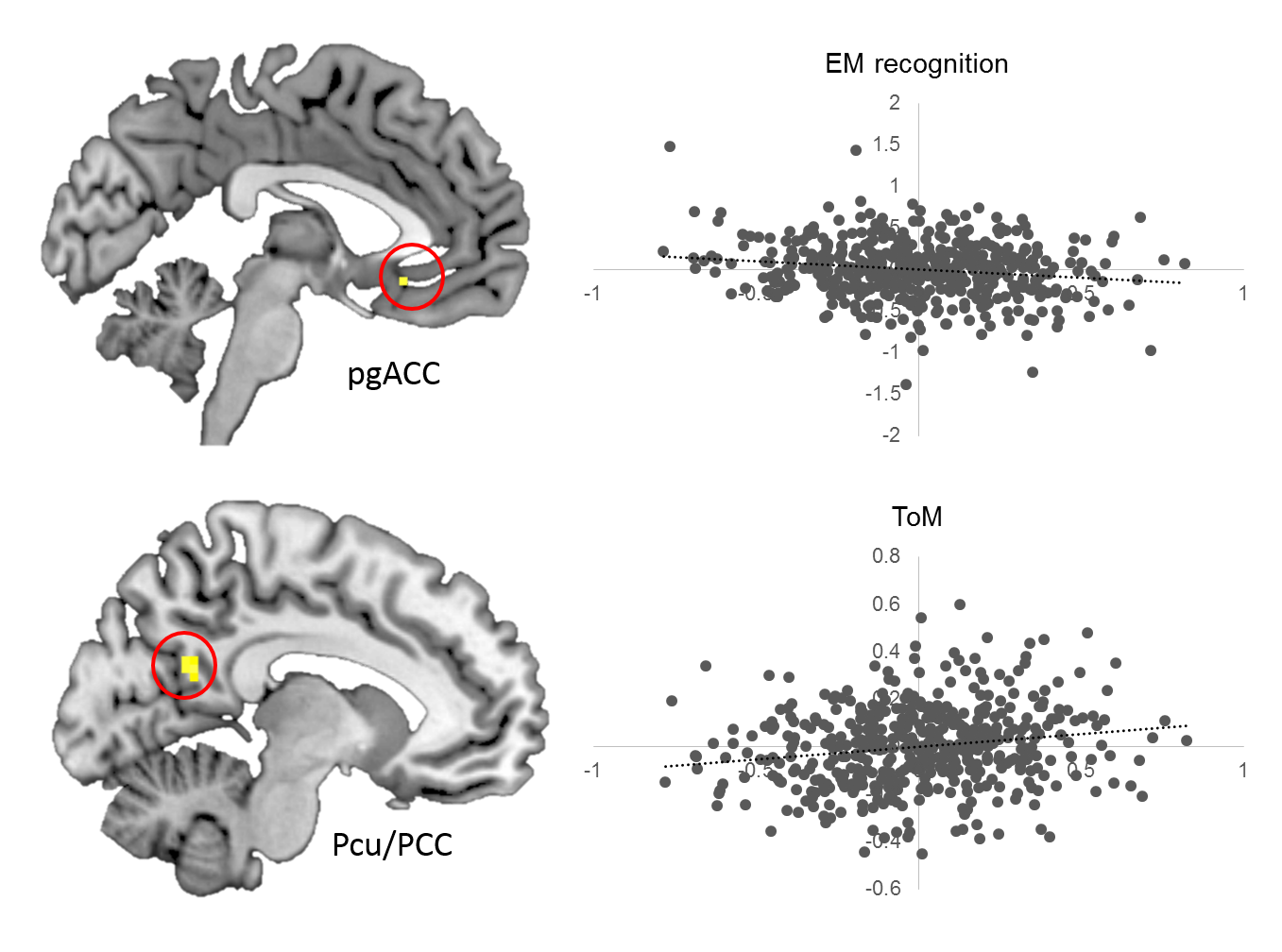


Results of association of risk profile scores (RPS) with intermediate phenotypes. Significant correlations were found for pgACC activation and individual RPS during EM recognition (x=-3, y=26, z=-11, F=13.72, Z=3.49, pFWE(ROI)=.047) and Pcu/PCC activation and individual RPS during mentalizing (x=-9, y=-55, z=22, F=13.90, Z=3.52, pFWE(ROI)=.025). x-axis = RPS, y-axis = size of effect.

**References**

1. Delaneau O, Marchini J, Zagury JF. A linear complexity phasing method for thousands of genomes. *Nature methods* 2012; **9**(2)**:** 179-181.

2. Howie B, Marchini J, Stephens M. Genotype imputation with thousands of genomes. *G3* 2011; **1**(6)**:** 457-470.

3. Purcell S, Neale B, Todd-Brown K, Thomas L, Ferreira MA, Bender D*, et al*. PLINK: a tool set for whole-genome association and population-based linkage analyses. *American journal of human genetics* 2007; **81**(3)**:** 559-575.

4. Devlin B, Roeder K. Genomic control for association studies. *Biometrics* 1999; **55**(4)**:** 997-1004.

5. Schizophrenia Working Group of the Psychiatric Genomics C. Biological insights from 108 schizophrenia-associated genetic loci. *Nature* 2014; **511**(7510)**:** 421-427.

6. Erk S, Meyer-Lindenberg A, Schnell K, Opitz von Boberfeld C, Esslinger C, Kirsch P*, et al*. Brain function in carriers of a genome-wide supported bipolar disorder variant. *Archives of general psychiatry* 2010; **67**(8)**:** 803-811.

7. Erk S, Meyer-Lindenberg A, Schmierer P, Grimm O, Tost H, Muhleisen T*, et al*. Functional impact of a recently identified quantitative trait locus for hippocampal volume with genome-wide support. *Translational psychiatry* 2013; **3:** e287.

8. Callicott JH, Bertolino A, Mattay VS, Langheim FJ, Duyn J, Coppola R*, et al*. Physiological dysfunction of the dorsolateral prefrontal cortex in schizophrenia revisited. *Cereb Cortex* 2000; **10**(11)**:** 1078-1092.

9. Grimm O, Heinz A, Walter H, Kirsch P, Erk S, Haddad L*, et al*. Striatal response to reward anticipation: evidence for a systems-level intermediate phenotype for schizophrenia. *JAMA psychiatry* 2014; **71**(5)**:** 531-539.

10. Mohnke S, Erk S, Schnell K, Schutz C, Romanczuk-Seiferth N, Grimm O*, et al*. Further evidence for the impact of a genome-wide-supported psychosis risk variant in ZNF804A on the Theory of Mind Network. *Neuropsychopharmacology : official publication of the American College of Neuropsychopharmacology* 2014; **39**(5)**:** 1196-1205.

11. Walter H, Schnell K, Erk S, Arnold C, Kirsch P, Esslinger C*, et al*. Effects of a genome-wide supported psychosis risk variant on neural activation during a theory-of-mind task. *Molecular psychiatry* 2011; **16**(4)**:** 462-470.

12. Schnell K, Bluschke S, Konradt B, Walter H. Functional relations of empathy and mentalizing: an fMRI study on the neural basis of cognitive empathy. *NeuroImage* 2011; **54**(2)**:** 1743-1754.

13. Hariri AR, Bookheimer SY, Mazziotta JC. Modulating emotional responses: effects of a neocortical network on the limbic system. *Neuroreport* 2000; **11**(1)**:** 43-48.

14. Van Overwalle F. Social cognition and the brain: a meta-analysis. *Human brain mapping* 2009; **30**(3)**:** 829-858.

15. Van Overwalle F, Baetens K. Understanding others' actions and goals by mirror and mentalizing systems: a meta-analysis. *NeuroImage* 2009; **48**(3)**:** 564-584.

16. Schubert R, Ritter P, Wustenberg T, Preuschhof C, Curio G, Sommer W*, et al*. Spatial attention related SEP amplitude modulations covary with BOLD signal in S1--a simultaneous EEG--fMRI study. *Cereb Cortex* 2008; **18**(11)**:** 2686-2700.

17. Turkeltaub PE, Eden GF, Jones KM, Zeffiro TA. Meta-analysis of the functional neuroanatomy of single-word reading: method and validation. *NeuroImage* 2002; **16**(3 Pt 1)**:** 765-780.

18. Nielsen FA, Hansen LK. Modeling of activation data in the BrainMap database: detection of outliers. *Human brain mapping* 2002; **15**(3)**:** 146-156.

19. Friedman L, Glover GH. Report on a multicenter fMRI quality assurance protocol. *Journal of magnetic resonance imaging : JMRI* 2006; **23**(6)**:** 827-839.
